# RelAp43, a Member of the NF-κB Family Involved in Innate Immune Response against Lyssavirus Infection

**DOI:** 10.1371/journal.ppat.1003060

**Published:** 2012-12-13

**Authors:** Sophie Luco, Olivier Delmas, Pierre-Olivier Vidalain, Frédéric Tangy, Robert Weil, Hervé Bourhy

**Affiliations:** 1 Institut Pasteur, Unité Dynamique des Lyssavirus et Adaptation à l'Hôte, Paris, France; 2 Université Paris Diderot, Sorbonne Paris Cité, Cellule Pasteur, Paris, France; 3 Institut Pasteur, Unité de Génomique virale et vaccination, Paris, France; 4 Institut Pasteur, Unité de Signalisation moléculaire et Activation cellulaire, Paris, France; Thomas Jefferson University, United States of America

## Abstract

NF-κB transcription factors are crucial for many cellular processes. NF-κB is activated by viral infections to induce expression of antiviral cytokines. Here, we identified a novel member of the human NF-κB family, denoted RelAp43, the nucleotide sequence of which contains several exons as well as an intron of the RelA gene. RelAp43 is expressed in all cell lines and tissues tested and exhibits all the properties of a NF-κB protein. Although its sequence does not include a transactivation domain, identifying it as a class I member of the NF-κB family, it is able to potentiate RelA-mediated transactivation and stabilize dimers comprising p50. Furthermore, RelAp43 stimulates the expression of HIAP1, IRF1, and IFN-β - three genes involved in cell immunity against viral infection. It is also targeted by the matrix protein of lyssaviruses, the agents of rabies, resulting in an inhibition of the NF-κB pathway. Taken together, our data provide the description of a novel functional member of the NF-κB family, which plays a key role in the induction of anti-viral innate immune response.

## Author Summary

The homeostasis of living cells is tightly regulated by signaling pathways, most of them being pleiotropic, which makes their understanding crucial in biology. One of them, the NF-κB pathway, includes a family of transcription factors involved in cell survival, proliferation, differentiation, and cell immunity. In this study, we identified a novel human member of the NF-κB family that we named RelAp43. It shares all the main characteristics of the already known NF-κB family members. Moreover, we demonstrated that RelAp43 induced specifically the expression of genes involved in the innate immune response against viruses. Interestingly, we showed that RelAp43 is specifically targeted by the matrix protein of rabies virus, which contributes to the pathogenesis of the virus and its escape from innate immune response. Taken together, our data provide the description of a novel functional member of the NF-κB family, which is involved in the induction of innate immune response against virus infection.

## Introduction

NF-κB proteins comprise a family of structurally-related eukaryotic transcription factors involved in the control of many physiological cellular processes [1]. This family contains five major Rel proteins in mammalian cells: p65/RelA, c-Rel, RelB, p50 and p52. All Rel proteins share the N-terminal homology domain (RHD) mediating homo- or hetero-dimerization, DNA binding, nuclear localization and interaction with the IκB proteins, the inhibitors of NF-κB. Only RelA, c-Rel and RelB have a transactivation domain (TAD) in their C-terminal region. In the vast majority of cell types, NF-κB is kept inactive in the cytoplasm through association with an inhibitory protein of the IκB family, which includes IκBα, IκBβ and IκBε, as well as p105 and p100, the cytoplasmic precursors of p50 and p52. Most of the signals that lead to activation of NF-κB, such as cytokines, various stress signals, and viral and bacterial infections, activate a high molecular weight complex containing a serine-specific IκB kinase (IKK). IKK is largely composed of three distinct subunits: the two related catalytic kinases - IKKα and IKKβ, and NEMO. Activated IKK then phosphorylates IκB on specific residues. Phosphorylated IκB are polyubiquitinated, then degraded by the proteasome machinery. As a consequence, free NF-κB dimers enter the nucleus and activate transcription of their target genes by binding specific DNA sequences named κB sites in the promoter region of numerous genes [2].

The list of target genes controlled by NF-κB is extensive and many are involved in key cellular processes such as cell survival, proliferation and immunity. The duration, strength and specificity of induction of these genes are tightly regulated [2]. Accumulating evidence suggests that alternative splicing events of NF-κB signaling components could be involved in controlling NF-κB signaling [3]. A variety of post-translational modifications of NF-κB constitute a second level of regulation [4]. Furthermore, non-Rel proteins that interact with NF-κB transcription factors within the nucleus constitute a third level of modulation [5]. These mechanisms of regulation of NF-κB activity notably impact the innate immune response. One of the major antiviral effectors induced by NF-κB are type I interferons, like interferon-β (IFN-β). RelA has been proposed to be crucial for early IFN-β expression that could prevent the replication of some RNA viruses [6]. RelA is also important for the maintenance of basal IFN-β expression in non-infected cells [7] that is important to prime a strong response in case of viral infection [8]. Viruses have evolved many strategies to manipulate the NF-κB pathway to their own benefit, especially to counteract the induction, signalling, or antiviral actions of the IFN circuit and to modulate cell death and apoptosis [9], [10]. Among RNA viruses, pathogenic strains of lyssavirus, the agent of rabies, have been shown to evade host innate immune response while non-pathogenic strains do not, indicating that response to infection is strain dependent [11].

Here, we describe a new splicing variant of RelA, which we named RelAp43, exhibiting all the properties of a NF-κB protein. Interestingly, RelAp43 is targeted by the matrix (M) protein of field but not vaccine strains of lyssaviruses, therefore blocking essential steps during activation of the innate immune response. The lyssavirus M protein is a small protein (∼20–25 kDa), forming oligomers that bind to the outside of the nucleocapsid, giving rigidity to the virion structure and providing a binding platform for viral glycoprotein trimers and the envelope membrane [12], [13]. It is also involved in a number of essential steps during infection and appears to issue multiple seemingly conflicting signals during the replication cycle of the virus [14]. Taken together, our data describe a novel functional member of the NF-κB family, which plays a key role in the induction of anti-viral innate immune response.

## Results

### RelAp43, a variant of RelA, is expressed in human cells

**Figure 1:**
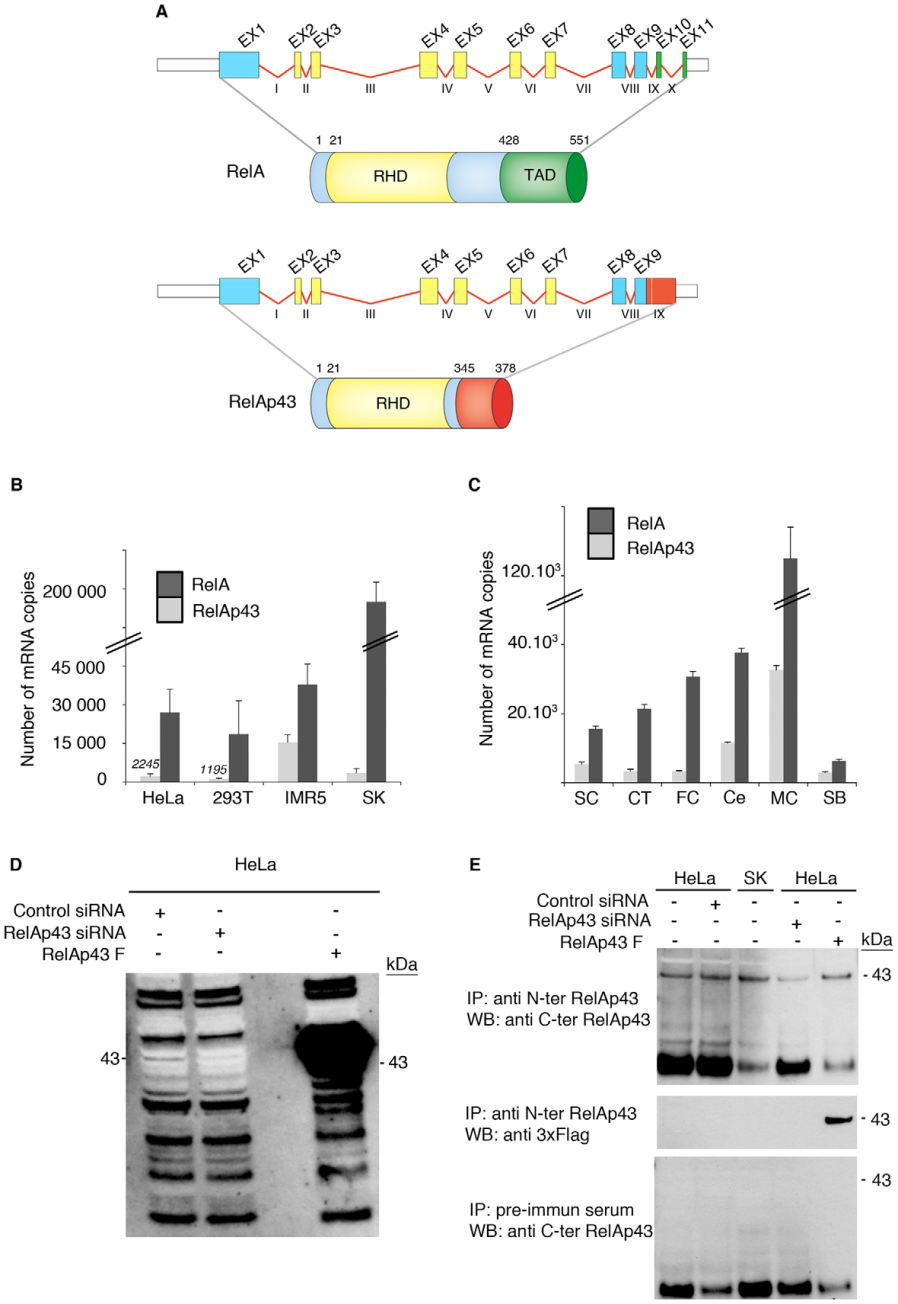
RelAp43 is a variant of RelA expressed in cell lines and tissues. (A) Schematic representations of RelA, RelAp43 and of the exons and introns present in their corresponding mRNA. Exons (EX on the Figure) are represented by boxes numbered with arabic numbers, and introns are indicated by red lines with roman numbers. While RelA protein is encoded by a mRNA composed of all 11 exons, a part of the intron IX (figured as a red box in the picture), followed by a stop codon, is found in the mRNA transcript encoding RelAp43. Rel Homology domains (RHD) of RelA and RelAp43 are depicted here, such as the transactivation domain (TAD) of RelA. (B) Absolute quantification of RelA- (black bars) and RelAp43- (grey bars) encoding mRNA in the indicated cell line. (C) Absolute quantification of RelA (black bars) and RelAp43 (grey bars) transcription in human primary tissues. SC: spinal cord; CT: cerebral trunk; FC: frontal cortex; Ce: cerebellum; MC: peripheral blood mononucleated cells; SB: skin biopsy. Results are the mean mRNA level obtained after 3 independent experiments. (D) Expression of RelAp43 in HeLa cells transfected either with a control siRNA, a specific siRNA directed against RelAp43 (RelAp43 siRNA) or overexpressing FLAG-tagged RelAp43 (RelAp43 F). Western blot analysis was performed with an antibody targeting RelAp43 C-terminal part on 50 µg of total cell lysates of each condition. See also Fig. S2. (E) Immunoprecipitation of RelAp43 in HeLa and SK-N-SH (SK) cells transfected either with a control siRNA, a specific siRNA directed against RelAp43 (RelAp43 siRNA) or overexpressing FLAG-tagged RelAp43 (RelAp43 F) or non-transfected. Immunoprecipitations were performed with a pre-immune serum or with antibody directed against RelAp43 N-terminal part then analyzed by western blot revealed either with an antibody targeting RelAp43 C-terminal part or with an anti 3xFLAG antibody. Only 30% of the sample corresponding to HeLa cells overexpressing RelAp43F was loaded on the gel in comparison with the other samples that were loaded in totality after immunoprecipitation.

Using the lyssavirus M protein as bait in a yeast two-hybrid screen against a human spleen cDNA library (Invitrogen), we found that it interacts with a protein initially assigned as RelA. After full sequencing of the cDNA of this M protein cellular partner, it appeared that it was a variant of RelA likely resulting from an alternative splicing event. Indeed, the 5′-end sequence of the cDNA of this variant aligned perfectly with the sequence of RelA mRNA (GeneBank refseq accession: NM_021975), while its 3′-end sequence corresponded to a part of the intron between exon 9 and 10 in the gene of RelA (Fig. 1A). The corresponding transcript encodes a protein of 378 amino acids with a calculated molecular weight of 43 kD which was accordingly named RelAp43. It contains the full RHD present in RelA but lacks its TAD, which is replaced by the short specific sequence of 33 amino acids (from amino acid 345 to 378) (Fig. 1A).

To check for the transcription of RelAp43 gene, its specific sequence corresponding to amino acids 345 to 378 was blasted to search for human mRNA or expressed sequence tags (EST). We found one mRNA (accession number: BC116830) isolated from a human pancreas epithelioid carcinoma that codes for RelAp43, and three human EST isolated from lung (DA58560), brain (AW054862) and prostate (DA873834) which have a sequence similar to the specific part of RelAp43, indicative of a potential expression of RelAp43 in various human organs. To confirm this hypothesis, we set up a real-time RT-PCR assay that amplified the respective specific parts of either RelAp43 or RelA mRNA. The quantification of the number of mRNA copies of RelA and RelAp43 was performed in several cell lines of human origin: HeLa cells, a cervical carcinoma cell line; 293T cells derived from embryonic kidney; SK-N-SH and IMR5, neuroblastoma cell lines. Even in low amount, RelAp43 gene transcription was detected in all the cell lines tested from about 1,100 copies in 293T cells to 15,000 in IMR5 cells per 100 ng of total RNA (Fig. 1B). As a control, we checked that no detection was noticed in the absence of reverse transcription (data not shown). The RelA/RelAp43 mRNA ratio is highly contingent upon the investigated cell type (from 2 in IMR5 cells, 10 in HeLa cells to 22 in 293T cells) further suggesting that the regulation of RelAp43 transcription is independent of that of RelA. As NFκB signaling is involved in many cellular pathways such as the balance between cell death and survival, inflammation, or the control in cell proliferation [15], [16], the expression level of RelAp43 could then be modified in immortalized cells compared to primary tissues. Therefore, we extracted total RNA from several normal human tissues and subjected them to quantification. We collected RNA samples from spinal cord, cerebral trunk, frontal cortex, cerebellum, peripheral blood mononucleated cells (PBMC) and skin biopsy. For each sample, we tested 100 ng of total RNA. As already shown, variations in the number of RelAp43 RNA copies (3,000 in skin biopsy to 32,000 in PBMC) (Fig. 1C) as well as in the RelA/RelAp43 mRNA ratio (2 in skin, 3 in PBMC, 9 in frontal cortex) were observed. To assess the expression of RelAp43, we performed a western blot analysis on 50 µg of total proteins from direct HeLa cell lysates after transfection with specific and control siRNAs, or FLAG-tagged RelAp43. We observed a 43 kD band, which intensity was reduced when specific siRNA was transfected (Fig. 1D, and Fig. S1). This specific siRNA did not affect the transcription of RelA mRNA (Fig. S1). The expression of RelAp43 was also observed in total cell lysates from HeLa and SK-N-SH after immunoprecipitation (IP) using an antibody targeting RelAp43 N-terminal part and western blot analysis using a specific antibody raised against the C-terminal extremity of RelAp43 (Fig. 1E).

Together, this demonstrates the transcription and the expression of a novel variant of RelA named RelAp43 in every cell line and tissue tested, such that it could be ubiquitous.

### RelAp43 can interact with members of the NF-κB family

**Figure 2:**
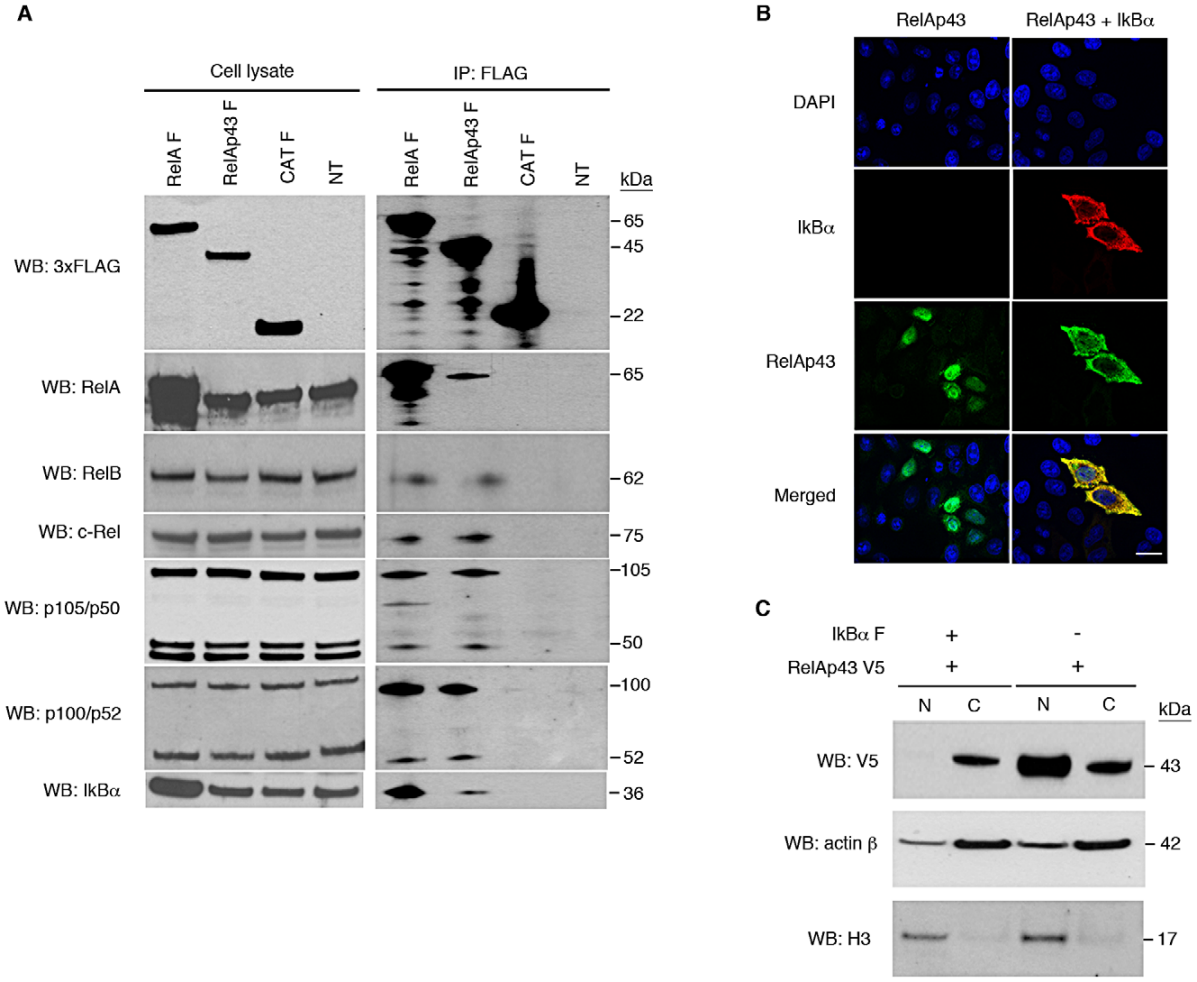
RelAp43 interacts with all human members of the NF-κB family and is supported by IκBα. All experiments were performed three times independently. (A) IP using anti-FLAG antibody of FLAG-tagged RelAp43 (RelAp43 F) or RelA (RelA F) or CAT (CAT F) expressing cells or non-transfected cells (NT). The presence of FLAG-tagged protein or endogenous transcription factors of the NF-κB family was analyzed by western blot using specific antibodies in cell lysates either before (left panel) or after IP (right panel). (B) Cellular localization of transfected RelAp43 (green) analyzed by immunofluorescence and apotome imaging in absence (left panel), or in presence of transfected IκBα (red, right panel). Nuclei were visualized using DAPI staining. The scale bar corresponds to 2 µm. (C) Western Blot analysis of nuclear (N) and cytosolic fractions (C) of RelAp43 V5 and IκBα F co-transfected cells after nuclear-cytosolic fractionation. Nuclear and cytosolic fractions were controlled using anti histone H3 and anti actin β antibodies, respectively. See also Figure S2.

To determine whether RelAp43 retains the ability to interact with the other proteins of the NF-κB family, FLAG-tagged RelAp43 was immunoprecipitated from transfected cells (HEK-293T and HeLa) and co-immunoprecipitation (co-IP) of endogenous members of the NF-κB pathway was analyzed by Western Blot (Fig. 2A). As expected, RelAp43 interacts with p105/p50, p100/p52, RelA, RelB and c-Rel. In addition, RelAp43 was found to interact with the NF-κB inhibitor IκBα. None of these partners were immunoprecipitated from non-transfected cells nor from FLAG-tagged CAT expressing cells, confirming the specificity of the co-IP (Fig. 2A). The same results were obtained with FLAG-tagged RelA, which indicates that RelAp43 has the same capacity of interaction with the other NF-κB family members as RelA (Fig. 2A). It is interesting to mention that the interaction of RelAp43 with RelA and IκBα was also demonstrated by yeast two-hybrid (data not shown).

### RelAp43 is retained in the cytoplasm by IκBα

To test whether RelAp43 could similarly to RelA enter the nucleus and whether its translocation could be inhibited by IκBα, we performed immunofluorescence experiments in transfected HeLa cells expressing V5-tagged RelAp43 alone or in association with 3xFLAG-tagged IκBα (Fig. 2B). Apotome analysis of the transfected cells indicates that in absence of IκBα, RelAp43 exhibits extensive colocalization with the DAPI staining of the nucleus, whereas in presence of IκBα, it is mostly cytoplasmic and co-localizes with IκBα (Fig. 2B). This was confirmed by nuclear cytosol fractionation experiments and analysis by Western Blot, which showed that RelAp43 was no more detectable in the nuclear fraction of cells co-transfected with IκBα (Fig. 2C). As a control, we checked that transfection of RelAp43 did not induce the production of IκBα (Fig. S2). Overexpression of RelAp43 could then overcome its retention by endogenous IκBα and be targeted in the nucleus. Thus, when IκBα was co-transfected with RelAp43, this induced the cytoplasmic retention of RelAp43. This suggests that RelAp43 can shuttle in different cell compartments notably via its interaction with IκBα (Fig. 2C).

### RelAp43 is a class I member of the NF-κB family

**Figure 3:**
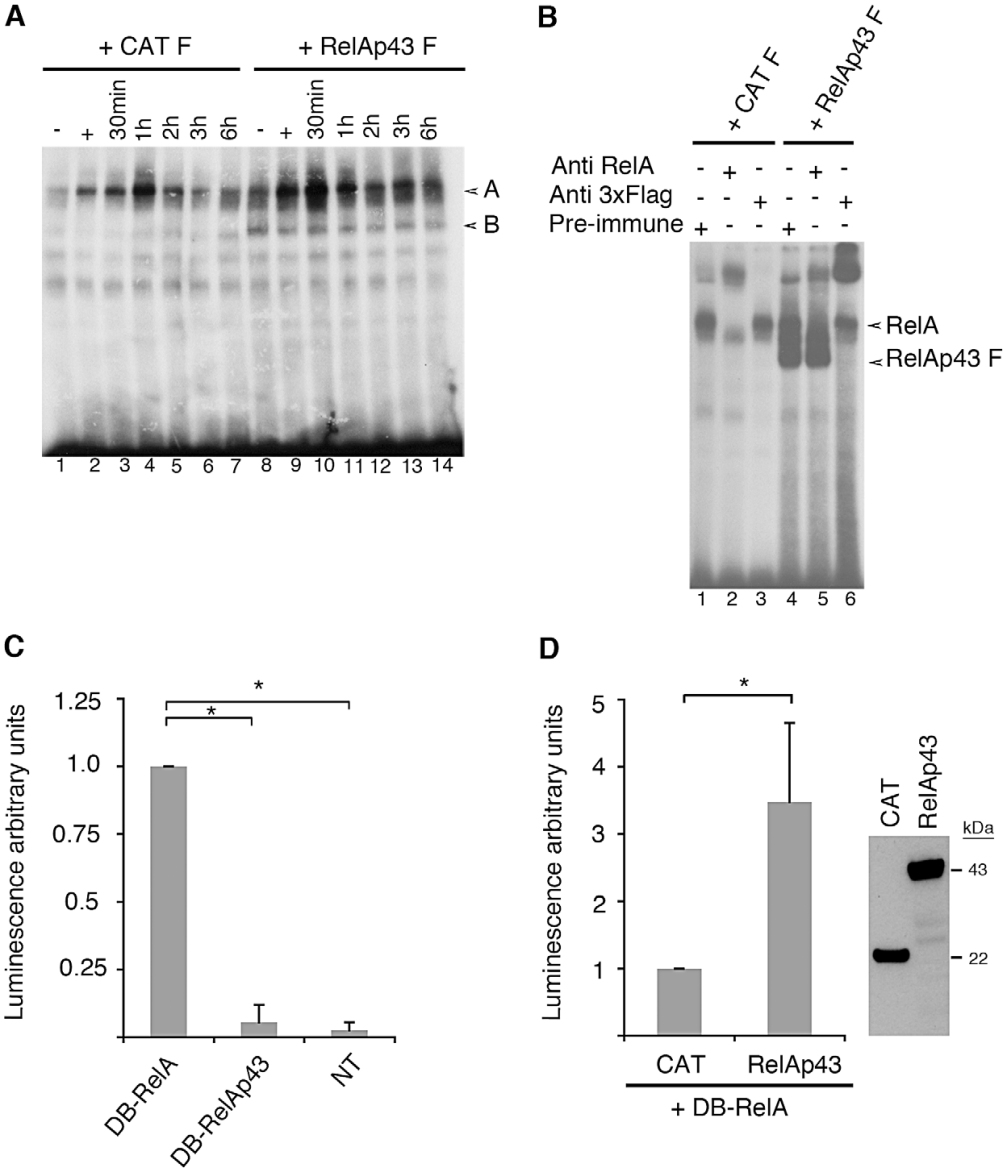
RelAp43 binds on DNA κB sites and potentiates RelA-mediated transactivation of gene expression. All experiments were performed three times independently. Error bars indicate standard deviations. *p<0.05. (A) EMSA analysis of nuclear extracts from FLAG tagged-CAT and FLAG tagged-RelAp43 transfected HeLa cells, using a radioactive κB probe. Cells were either left untreated (−, lanes 1 and 8), treated for 15 min with 10 ng/mL TNF-α (+, lanes 2 and 9) or treated for 15 min with 10 ng/mL TNF-α followed by chase periods of 30 min to 6 hours (lanes 3–7 and 10–14). Band A: RelA bound to κB-probe; band B: RelAp43 bound to κB-probe, as demonstrated in Figure 3B. (B) Supershift analysis of the complexes bound to the κB-probe. 100 ng of α-RelA antibody, α-FLAG antibody or pre-immune serum was incubated with the EMSA reaction mixture before gel electrophoresis. (C) Measurement of the luminescence of cells expressing luciferase under control of the Gal4 promoter and RelA or RelAp43 fused to the Gal4 DNA Binding domain (named as DB-RelA or DB-RelAp43 on the figure, respectively) or non transfected (NT). DB-RelA was arbitrary set to 1. (D) Luciferase assay of RelA transactivation properties in presence of CAT or RelAp43. RelAp43- or CAT-encoding plasmids were added to the transfection mix in the same conditions as in (C) (see also Figure S3). Their expression level was controlled by the western blot analysis showed on the right of the Figure. The level of luminescence obtained in the presence of DB-RelA and CAT encoding plasmids was arbitrary set to 1.

To assess that RelAp43 could associate with DNA and interfere with DNA-bound NF-κB complexes, we performed electrophoresis mobility assays (EMSA) experiments and supershift experiments using an anti-FLAG antibody on nuclear fractions of unstimulated or TNF-α-induced HeLa cells expressing either FLAG-tagged CAT or FLAG-tagged RelAp43. While we could not detect nuclear NF-κB DNA-binding activity in unstimulated control HeLa cells (Fig. 3A, lane 1), this was strongly induced after the addition of TNF-α for 15 min (Fig. 3A, band A, lane 2). When the cytokine was removed, NF-κB activity started decreasing following a chase period of 2 hours (Fig. 3A lane 5). Interestingly, a higher basal NF-κB activation was observed following transient expression of RelAp43 (Fig. 3A lane 8) compared to CAT (Fig. 3A lane 1). NF-κB activation was potentiated by treatment with TNF-α (Fig. 3A lane 9). During the chase period, RelAp43 sustained NF-κB DNA-binding activity (Fig. 3A lanes 10–14) to a higher level and for a longer period than CAT (Fig. 3A lanes 3–7). This result probably indicates that RelAp43 sustained the interaction between endogenous NF-κB in HeLa cells and the κB probe. Incubation of nuclear fractions of RelAp43-transfected cells (Fig. 3B), with anti-RelA antibody which recognizes the unique C-terminus part of RelA, supershifted the complex to a higher molecular weight (Fig. 3B lane 5) similar to that observed in CAT- expressing cells (Fig. 3B lane 2), whereas the migration of this complex was not modified by pre-immune serum (Fig. 3B lanes 1 and 4). Moreover, an additional band that migrates faster appeared in FLAG-tagged RelAp43 compared to FLAG-tagged CAT expressing cells (Fig. 3A band B, lanes 8–14), suggesting some interaction between FLAG-tagged RelAp43 and the κB probe. In agreement with this hypothesis, this band was supershifted when the nuclear extract of RelAp43-transfected cells was incubated with anti-FLAG antibody (Fig. 3B lane 6) but not following incubation with anti RelA antibody (Fig. 3B lane 5). Although we could not conclude about the partners of RelAp43 from the supershift experiments, they did clearly indicate that a complex containing RelAp43 was associated with κB probe. This was not a tag artifact since FLAG-tagged CAT was not able to associate with the same probe than FLAG-tagged RelAp43.

To test whether RelAp43 presents a transactivation activity while its sequence differs from that of RelA on its C-terminal extremity, we cloned it in fusion with the DNA binding domain of Gal4 (DB), a yeast modular transcription factor, and measured the expression of firefly luciferase gene under control of a promoter sequence containing binding sites for Gal4-DB. The same experiment was performed with RelA as a positive control. As expected, DB-RelA induced a strong expression of the luciferase gene under control of Gal4 promoter. In contrast, DB-RelAp43 did not induce expression of luciferase gene under control of Gal4 promoter since the luminescence measured was not significantly different from that of untransfected cells (Fig. 3C). Thus and as expected considering its sequence, RelAp43, like p50 or p52, contains a full RHD but lacks TAD even in its C-terminal specific extremity of 33 amino acids. Since RelAp43 interacts with RelA (as shown by co-IP, see Fig. 2A), we co-transfected DB-RelA with RelAp43 or CAT as a control to test the effect of RelAp43 on DB-RelA dependant induction of luciferase gene and checked the level of expression of RelAp43 and CAT (Fig. 3D). Increasing doses of RelAp43 co-expressed with constant amounts of DB-RelA, clearly indicated a stimulating role of RelAp43 (Fig. S3A and S3B). We then used 160 ng of CAT- and RelAp43-encoding plasmids for transfection as this dose exhibited the best trade-off between the intensity and the variability of the luminescence signal (Fig. S3A). In that case, the co-expression of CAT had no effect on DB-RelA-dependant luciferase expression, whereas the co-expression of RelAp43 increased the luciferase expression more than 3 fold (Fig. 3D). Overall, these results indicate that RelAp43 is a class I member of the NF-κB family which can potentiate the transactivation potential of RelA.

### RelAp43 modifies the equilibrium of p50-comprising dimers and potentiates the transcription of several NF-κB dependent genes

**Figure 4:**
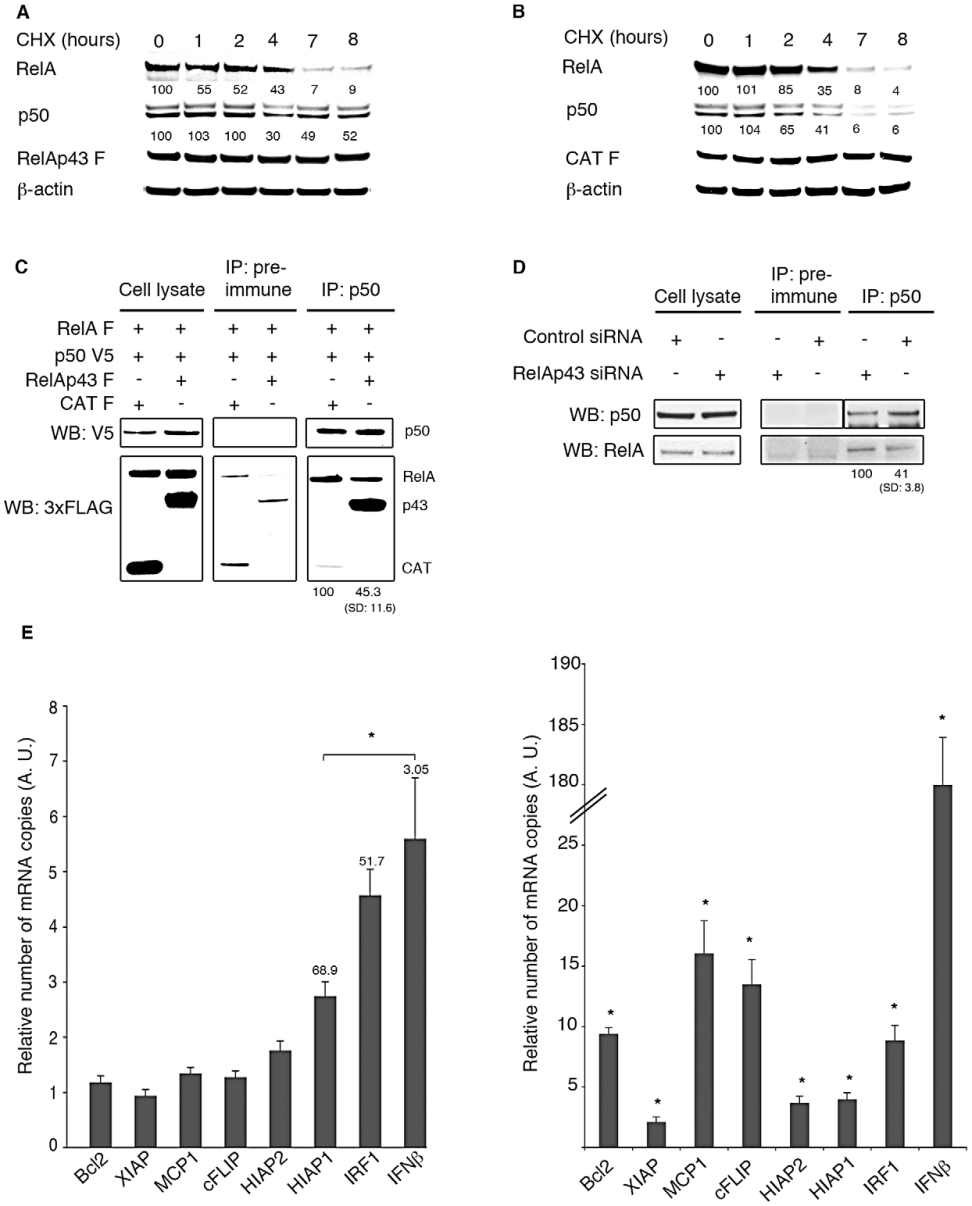
RelAp43 modifies the equilibrium of p50-comprising dimers and specifically potentiates the transcription of several NF-κB dependent genes. Analysis of the stability of RelA and p50 using cycloheximide blockage of protein translation in HeLa cells overexpressing RelAp43 (A) or CAT (B). Quantification of the RelA and p50 expression, corrected by the actin controls, are given under each RelA and p50 bands. Expression levels in cells not treated with cycloheximide (time 0) were used as controls and set up to 100. (C) Modulation of transfected RelA-p50 dimer formation according to the amount of RelAp43. Dimer formation involving transfected RelA and p50 was analyzed by co-IP using anti-p50 antibody on protein extracts of HeLa cells cotransfected by FLAG-tagged RelA, V5-tagged p50 and FLAG-tagged RelAp43 or CAT as a control. The amount of FLAG-tagged RelA interacting with V5-tagged p50 was assessed using anti-FLAG antibodies. The quantification levels presented at the bottom of the lanes are the means and standard deviations (SD) of the intensity of the band corresponding to RelA obtained by substracting the intensity of the band observed with the pre-immune serum to the one obtained after p50 immunoprecipitation. These experiments (A, B and C) were repeated 3 times independently and results presented are representative of the three repetitions. (D) Modulation of endogenous RelA-p50 dimer formation according to the amount of endogenous RelAp43. Quantification of RelA obtained after co-IP using anti-p50 antibody (IP: p50) was corrected by that observed using a pre-immune serum (IP: pre-immune). Expression levels in cells transfected with control siRNA were used as controls and set up to 100. The experiments were repeated twice independently and the quantification levels presented at the bottom of the lanes are the means and standard deviations (SD) obtained. (E) Relative level of transcription of a set of apoptosis genes in HeLa cells transfected with RelAp43- or CAT-encoding plasmids (on the left) and with RelA- or CAT- encoding plasmids (on the right). The levels of RelAp43 and RelA mRNAs were normalized to the level of GAPDH mRNA and reported relatively to the level measured in CAT-expressing cells used as control (set to 1, not figured). Results presented here are the mean of three independent experiments. For one given repetition, all eight genes were studied on the same cDNAs using specific set of primers. The percentage of induction of *HIAP1, IRF1* and *IFN-β* mediated by RelAp43 and relatively to that mediated by RelA is indicated on the corresponding bars (on the left). *p<0.05.

Dimerization is required for NF-κB binding to DNA and 15 homo- and heterodimers have so far been described. We hypothetized that RelAp43, as a class I NF-κB protein, could modify the equilibrium between the different combinations of NF-κB dimers. As active NF-κB complexes are mainly composed of RelA-p50 dimers, we analyzed the stability of these dimers in response to cycloheximide, a protein synthesis inhibitor, in RelAp43- or CAT-transfected cells (used as control cells). RelA decay was identical in CAT- and RelAp43-transfected cells. Most interestingly, the expression of RelAp43 (Fig. 4A) but not that of CAT (used as a control) (Fig. 4B) prevented p50 degradation. We then investigated the propensity of RelAp43 to modulate the formation of p50-RelA dimers by co-IP experiments using an anti-p50 monoclonal antibody. In cells transfected with p50 and RelA, RelAp43 but not CAT overexpression affected the formation of RelA-p50 dimers (Fig. 4C). To confirm if this modulation exerted by RelAp43 in transfected cells could also be seen in more physiological conditions, we performed the same type of experiment in cells partially depleted for RelAp43 by specific siRNA (52%; see Fig. S1 and Fig. 4D). Following this depletion, we observed that the amount of endogenous RelA involved in dimer formation with p50 was higher than in normal conditions (Fig. 4D).

Therefore, the selective stabilization of different members of the NF-κB family mediated by RelAp43 could influence their relative amounts in the nucleus and their interaction with DNA. To explore the impact of RelAp43 on genes expression, we focused on eight genes belonging to the apoptosis pathway and innate immune response, in which NF-κB signaling is known to be involved. To this aim, we designed RT-PCR sets of primers to quantify the transcription of *Bcl2*, *XIAP*, *MCP1*, *cFLIP*, *HIAP1*, *HIAP2*, *IRF1* and *IFN-β* genes in HeLa cells overexpressing RelAp43 or RelA for 48 h, compared to HeLa cells overexpressing CAT for 48 h. For five of these genes, transcription levels were not significantly different when RelAp43 was overexpressed compared to those observed with CAT (Fig. 4E). In contrast, the transcription of *HIAP1*, *IRF1* and *IFN-β* genes was significantly enhanced in the presence of RelAp43 (Fig. 4E, left panel). As expected, transfection of RelA, which activates by itself NF-κB pathway, significantly induced the transcription of all eight genes since they are all known to respond to NF-κB (Fig. 4E, right panel). HIAP1, IRF1 and IFN-β induction mediated by RelAp43 represented respectively 68.9%, 51.7% and 3.05% of the induction mediated by RelA, further suggesting that RelAp43 induces a gene modulation, probably through its association with class II NF-κB family members. Furthermore, the observed pattern is different of that mediated by RelA in terms of target genes and intensity of their transcription.

Collectively these data indicate that RelAp43 is preferentially involved in dimer formation with p50 thereby limiting the number of RelA-p50 dimers. Furthermore, RelAp43 increases the expression of NF-κB target genes like *HIAP1*, *IRF1* and *IFN-β* in a specific manner compared to RelA.

### Lyssavirus M proteins from field isolates interact with RelAp43 and inhibit NF-κB signaling

**Figure 5:**
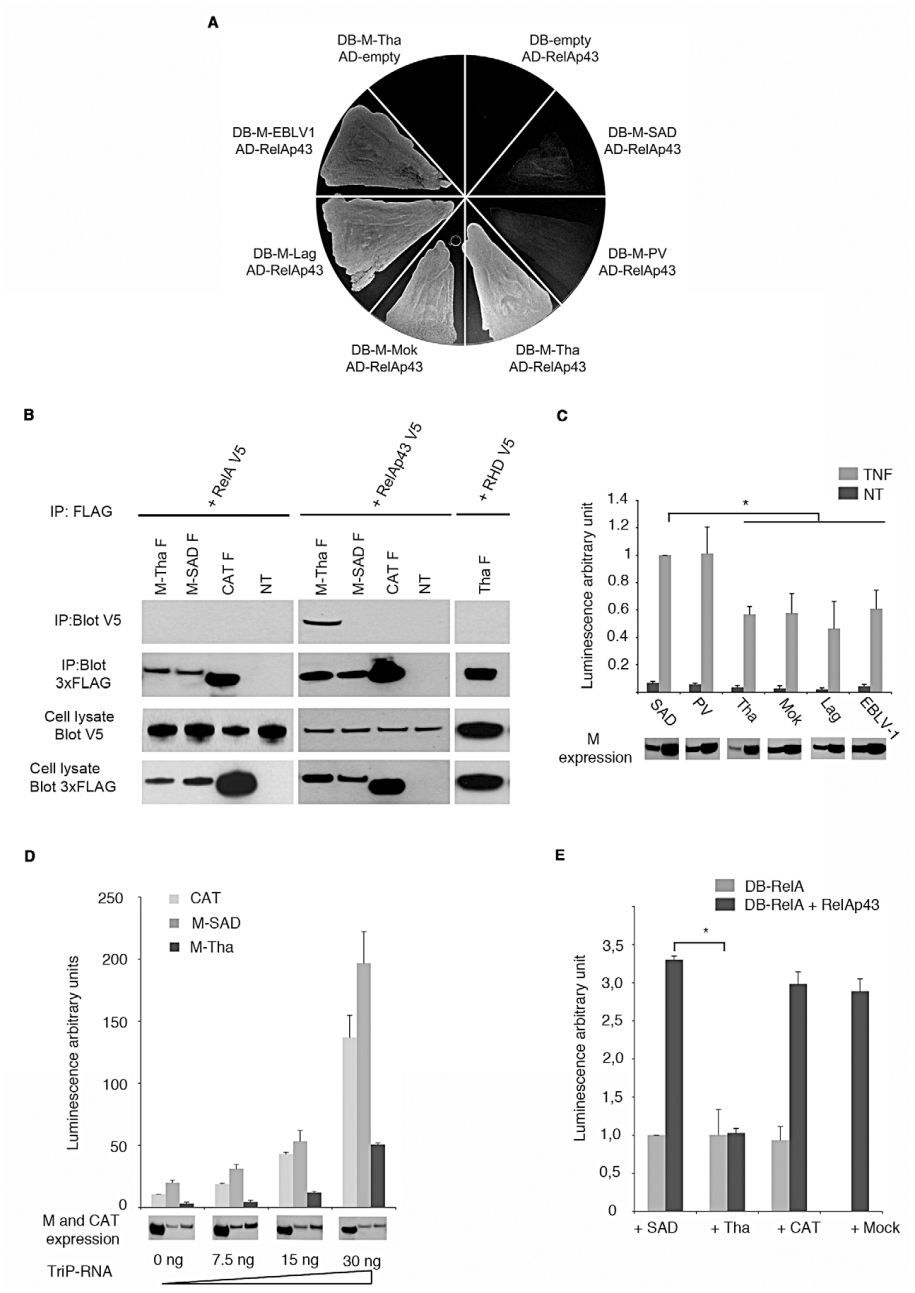
Specific interaction of RelAp43 with the M protein of rabies virus and inhibition of NF-κB pathway. The results presented here are representative of three independent experiments. Error bars indicate standard deviations. *p<0.05. (A) Determination of RelAp43 interaction with various lyssavirus M proteins in the yeast two-hybrid system. Yeast cells expressing Gal4-DB fused to M proteins from tested lyssaviruses were co-transformed to express Gal4-AD fused to RelAp43. Cells were plated on synthetic medium lacking histidine so that yeast growth is determined by M interaction with RelAp43 and activation of HIS3 reporter gene. (B) Western blot analysis of co-IP involving M proteins and RelA or RelAp43. Co-IP was performed using anti-FLAG antibody on cells co-transfected with on the one hand plasmid encoding RelA V5, RelAp43 V5 or the common part between them (RHDb) and on the other hand plasmid encoding FLAG-tagged M-Tha, M-SAD, CAT or non-transfected cells (NT). IP of FLAG-tagged proteins (not figured) and co-purification of V5-tagged protein were assessed (WB:V5 on the figure). Initial cell lysates were checked for V5- (cell lysate blot V5) and FLAG-tagged (cell lysate blot 3xFLAG) proteins expression. (C) Modulation of NF-κB activation in the presence of M protein from different lyssavirus strains after short-term stimulation. The NF-κB pathway was exogenously activated using 10 ng/mL TNF-α during 5 h (grey bars) or left untreated (black bars). The M protein of vaccinal strain SAD-B19 was arbitrary considered as a reference. The expression levels of M proteins in each condition were assessed by western blot (bottom of the figure). (D) Modulation of NF-κB activation in the presence of M-Tha (black bars), M-SAD (dark grey bars) or CAT (light grey bars) after extended stimulation. Increasing amounts of tri-phosphate RNA mimicking viral infection during 24 h were used. The expression levels of M-Tha, M-SAD and CAT in each condition were assessed by western blot (bottom of the figure). (E) Luciferase assay of RelA transactivation properties either without any other plasmid (light grey bars) or with a plasmid encoding RelAp43 (dark grey bars) plus a plasmid coding for the indicated M protein (SAD, Tha) or CAT or no additional plasmid (Mock).

Next we investigated the role of RelAp43 during viral infection. We first determine the interaction of various M proteins from different lyssaviruses with RelAp43. In the yeast two-hybrid system, we found that M proteins of field isolates of four species of lyssavirus (M-Tha, M-Mok, M-Lag, M-EBLV-1) could interact with RelAp43, whereas M proteins from laboratory strains or vaccine strains (M-PV and M-SAD) did not (Fig. 5A). To validate these results, we performed co-IP experiments (Fig. 5B). In agreement with two-hybrid screen, we observed that IP of FLAG-tagged M-Tha but not of M-SAD led to the recovery of V5-tagged RelAp43. The specificity of the interaction of M-Tha with RelAp43 was further confirmed since neither FLAG-tagged CAT expressing cells nor control cells that did not express any FLAG-tagged protein (NT) allowed the precipitation of RelAp43 with a FLAG antibody. Moreover, this interaction was specific to RelAp43 since neither RelA nor the common part of RelA and RelAp43 encompassing the RHD (RHDb in Fig. 5B) could interact with M-Tha in the same conditions. This result suggests that the unique region of RelAp43 is necessary for the interaction with M-Tha. However, since we could not express efficiently the unique C-terminal 33 amino acids of RelAp43, we could not formally demonstrate that this region is involved in the interaction with M-Tha. Since RelAp43 was targeted by lyssavirus M we tested whether infection by lyssavirus could modulate RelAp43 transcription. The transcription of RelAp43 was increased from 2 to 3.5 fold at 48 h p.i. compared to cells infected for only 1 h (Fig. S4).

To test whether M proteins from field isolates but not from vaccine strains could modulate NF-κB pathway, we used a luciferase reporter gene under control of κB sites. Interestingly, cells expressing M-Tha, M-Mok, M-Lag and M-EBLV-1 which all interact with RelAp43 showed a reduced NF-κB-dependent luciferase activity in response to TNF-α (5 h) compared to M-SAD or M-PV expressing cells which do not target RelAp43 (Fig. 5C). To further investigate the modulation of NF-κB pathway later in infection, cells were transfected with increasing amounts of 5′-triphosphate RNA and cultured during 24 hours. Indeed, 5′-triphosphate RNA is a virus-associated molecular pattern that triggers antiviral responses, including NF-κB activation. In this latter condition, a clear inhibitory effect of M-Tha compared to M-SAD and CAT (used as a control) was observed (Fig. 5D).

To further understand the functional interplay between M proteins and RelAp43, M-Tha and M-SAD were coexpressed with RelAp43 and tested on the previously described RelA-mediated transcription luciferase assay. Our results indicate that DB-RelA alone (RelA fused to Gal4 DNA binding domain) induced expression of luciferase under control of Gal4 promoter and that CAT, M-SAD and M-Tha had no effect on this basal level (Fig. 5E). Interestingly, when RelAp43 is added to DB-RelA, which was previously shown to induce RelA-mediated transcription of luciferase (see Fig. 3D), the level of luciferase recovered was significantly decreased in the presence of M-Tha only (Fig. 5E). This further confirms that RelA-mediated transcription is specifically modulated by interaction between M-Tha and RelAp43 and is probably involved in M-mediated inhibition of NF-κB pathway.

### RelAp43 is involved in IFN-β transcription during lyssavirus infection

**Figure 6:**
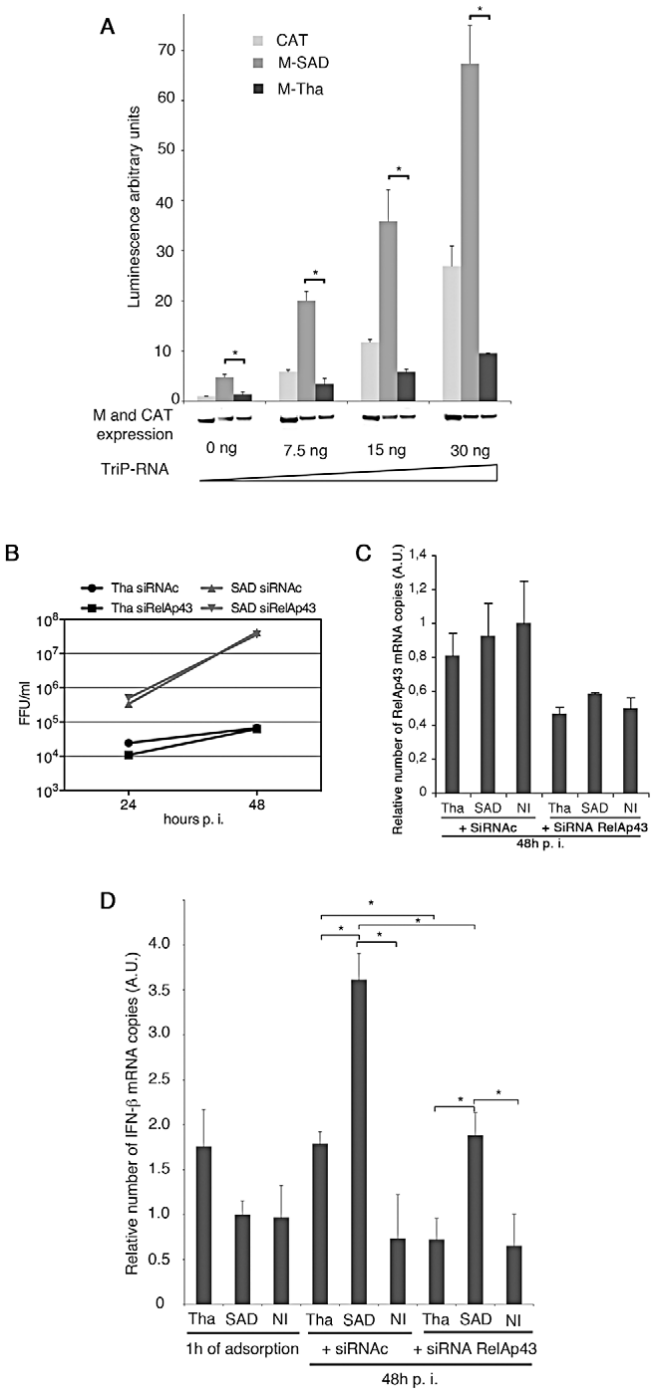
RelAp43 is involved in IFN-β transcription during lyssavirus infection. The results presented are the means of five (A) or three (B, C, D) independent experiments. Error bars indicate standard deviations. *p<0.05. (A) Luciferase assay of *IFN-β* promoter activation in presence of M protein. M-Tha (black bars), M-SAD (dark grey bars), or CAT (light grey bars) as a control were used. The measure obtained with M-SAD was arbitrary set to 1. The expression levels of M-Tha, M-SAD and CAT in each condition were assessed by western blot (bottom of the figure). (B) Infectious titres of Tha and SAD viruses from supernatant of Hela cells infected at an MOI of 1 at 24 and 48 hours p. i. in the presence of siRNA control (siRNAc) or anti-RelAp43 siRNA (siRelAp43). Titers are given in Fluorescent focus units per ml (FFU/ml). (C) Decrease of the number of RelAp43 mRNA copies in Hela cells infected with Tha or SAD virus and in non-infected cells (NI) in the presence of anti-RelAp43 siRNA (siRNA RelAp43) compared to siRNA control (siRNAc) and measured by quantitative RT-PCR. The level of transcription of RelAp43 in Hela cells measured 48 hours after infection with SAD and in the presence of control siRNA was arbitrary set to 1. (D) Modulation of IFN-β mRNA levels detected by quantitative RT-PCR in HeLa cells transfected by either a control siRNA or an anti RelAp43 siRNA, and infected with Tha or SAD virus compared to non-infected cells (NI).

Since RelAp43 appears to be involved in IFN-β pathway (Fig. 4E), we wanted to correlate the induction of IFN-β expression by different strains of lyssavirus to the ability of their M protein to interact with RelAp43. To this aim, we first used an *in vitro* assay system to measure luciferase activity under control of IFN-β promoter in cells expressing M-Tha, M-SAD or CAT used as a control (Fig. 6A). IFN-β pathway was stimulated in these cells using different amounts of 5′-triphosphate RNA molecules. A significant difference in the level of luciferase activity following the expression of M-Tha compared to M-SAD or CAT was observed. The activation of *IFN-β* promotor was strongly reduced in cells expressing M-Tha, compared to other transfected cells, especially at the highest dose of 5′-triphosphate RNA (Fig. 6A). Thus, the expression of M-Tha alone was sufficient to inhibit IFN-β transcription.

To better understand the importance of RelAp43 in the induction of IFN-β transcription after viral infection, the expression of RelAp43 was silenced using specific RNAi (Fig. 6B and 6C). Knock-down of RelAp43 was similar (nearly 40 to 50% of depletion) in infected cells at 48 h post infection (p.i.) and in non-infected cells analyzed at the same time (Fig. 6C). After infection at an MOI of 1, infectious supernatant virus production of the vaccine strain SAD (highly adapted to cell culture) reached approximately 5.10^5^ and 4.10^8^ fluorescent forming units (FFU)/ml at day 1 and 2 p.i., respectively (Fig. 6B). As expected, the field isolate Tha virus, showed a marked delay in virus production and yielded slightly less than 10^5^ FFU/ml at day 2 p.i. The transfection of a specific anti RelAp43 siRNA or a control siRNA did not modified the production of any of the two viruses (Fig. 6B). The transcription of IFN-β using real-time quantitative PCR was studied at 48 h p.i.. As expected, in infected cells transfected with the control RNAi, the level of transcription of IFN-β was increased when compared with non-infected cells (Fig. 6D). When RelAp43 expression was turned down, IFN-β mRNA level after Tha- or SAD- infection was half-reduced whereas it was not modified in non-infected cells.

In Tha infected cells, IFN-β mRNA level even reached the same level than in non-infected cells in the presence of anti-RelAp43 siRNA, thus further demonstrating a role for RelAp43 in IFN-β transcription cascade during virus infection. The remaining difference in the induction of IFN-β between Tha and SAD when cells are transfected with anti-RelAp43 siRNA may be due to the fact that the expression of RelAp43 was not completely knocked down (Fig. 6C), and that the interaction between M-Tha and the remaining RelAp43 further inhibited the residual RelAp43-mediated IFN-β gene transcription compared to SAD. However, other explanations may also be considered. In particular, it could not be ruled out that the effect could be partly due to the differences in virus titers. Further, other components than the M protein in SAD virus may play a role in an RelAp43-independant IFN-β gene transcription or that higher SAD replication allowed higher IFN-β mRNA level.

Altogether these results demonstrate that IFN-β response is modulated by the ability of M proteins to bind RelAp43 and that RelAp43 is involved in the IFN-β response to lyssavirus infection.

## Discussion

The activity of NF-κB transcription factors is tightly regulated by the cell dimer repertoire [17]. Homeostasis of the cell is thus clearly related to the association and dissociation of each of the 15 monomer pairs, and this process could be even more complex if the NF-κB included additional transcription factors. In this context, the characterization of alternative splicing events leading to the translation of additional members of NF-κB family [3] is paramount to understand the regulation of this signaling pathway. The current study has identified a new RelA variant with a molecular weight of 43 kD - RelAp43. It shares with RelA a full and active RHD, but lacks the TAD that is replaced by a specific domain encoded by an intron sequence located between exons 9 and 10 of the human *relA* gene [18]. This sequence of 33 amino acids does not show any transcription activation activity; thus RelAp43 seems to be a new class I member of the Rel protein family. Therefore, the structure of the RelAp43 gene differs from that of 3 previously described splicing variants of RelA (p65Δ, p65Δ2, and p65Δ3) which strictly result from deletion in RelA sequence coding for the RHD [19], [20], [21], [22]. The most studied of these splice variants, p65Δ, looses its association with p50, presents a weak association with p65, and a reduced ability to bind DNA. The identification of RelAp43 further illustrates the potential role of alternative splicing or truncated forms in the NF-κB signaling pathways as already demonstrated in a mice model after injury [23] and during *Leishmania* infection [24].

**Figure 7:**
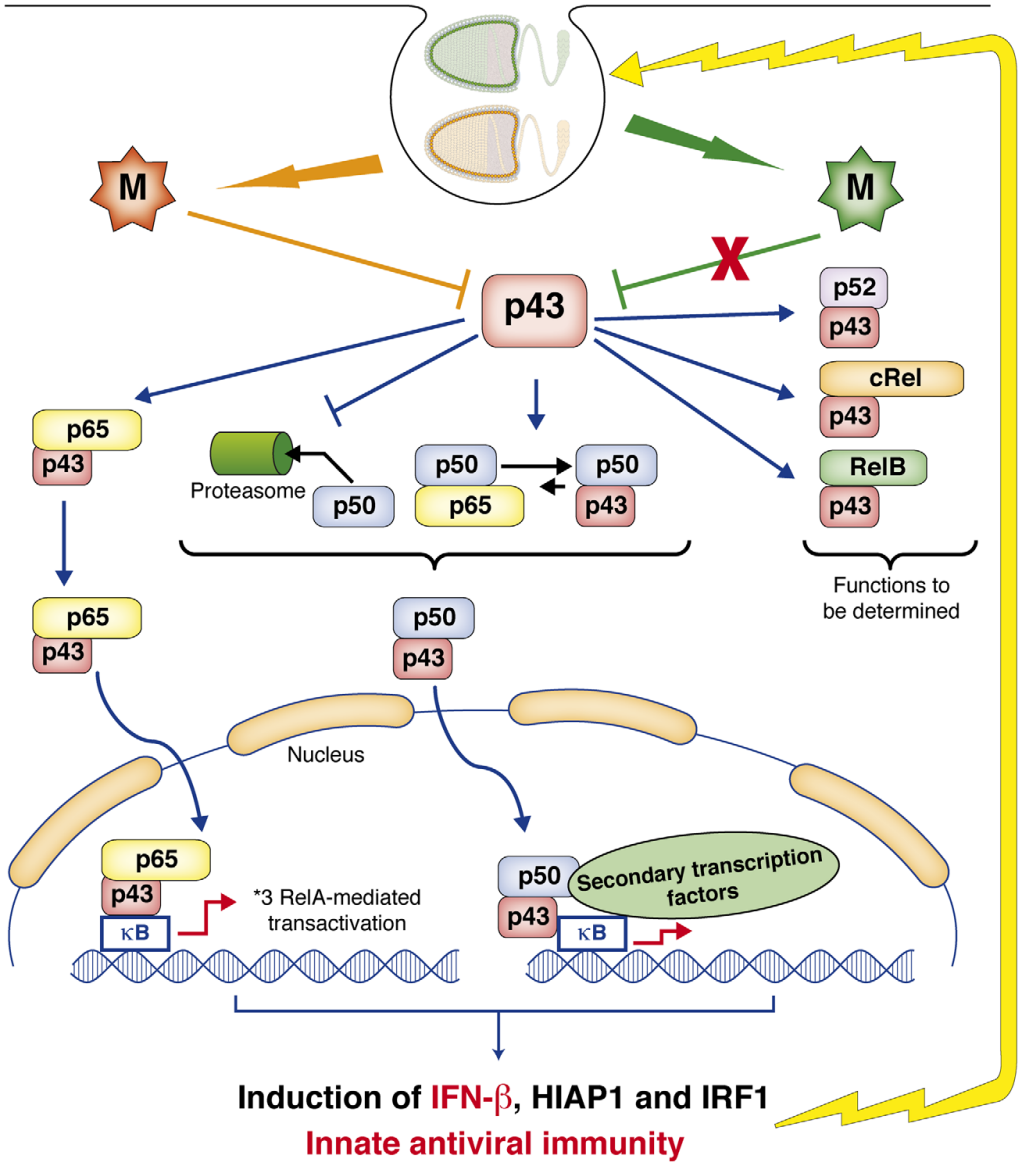
Proposed model for the role of RelAp43 and of the M protein of lyssavirus in the regulation of the NF-κB dimer repertoire and downstream action on the transcription of different genes involved in innate immune response. RelAp43 is designed as p43, M protein of lyssavirus wild isolates in orange; laboratory adapted and vaccine strains in green.

RelAp43 mRNA is present in all tissues and cell lines tested, although its amount is about 10 times less than that of RelA. This indicates that NF-κB dimers including RelAp43 should belong to the common dimer repertoire of human cells. However, the different mRNA ratios of RelA and RelAp43 observed in different cell lines and tissues indicate some variations in these repertoires as reported previously for RelA [21] and other RHD polypeptide synthesis [2]. How this new member of the Rel family interferes with NF-κB dimer generation and with transient immunological stimuli remains a crucial question. RelAp43 retains all activities related to dimer formation and DNA binding through its RHD domain. As suggested by the co-IP experiments, RelAp43 acts as a binding partner for cRel, RelB, RelA, p50, p52, p100 and p105 as previously demonstrated for p50 and p52, two other RHD polypeptides that also lack TAD. Therefore, the formation and the stability of the different NF-κB dimers including those with RelAp43 may depend on the level of expression of RelAp43 and on the capacity of the different types of dimers to associate and dissociate [25]. The action of RelAp43 on the dimer equilibrium could result from several processes (Fig. 7). First and as for RelA, RelAp43 function could be regulated by the IκBs as indicated by the ability of RelAp43 to interact with IκBα and by the fact that overexpression of IκBα re-localized RelAp43 to the cytoplasm. RelAp43 could not only modulate the formation of active NF-κB dimers, but be also part of NF-κB dimers that bind to DNA through κB sites and induce transcription [26], [27] as indicated by the EMSA experiments showing a stabilization of the RelA complex by RelAp43 in response to TNF-α stimulation. Furthermore, it remains possible that RelAp43 may also act in conjunction with enhanceosome at distant sites for a complete activation of certain NF-κB responsive genes. This has been shown for the *IFN-β* gene for which a cluster of κB sites 3′ downstream to the gene is also needed for a maximal gene expression after LPS treatment [28]. Finally, RelAp43 could form new transcription factor(s) in association with other members of the Rel protein family, as suggested by the co-IP experiments that show interactions with the other members of the NF-κB family. Some of the NF-κB dimers, and especially those including RelAp43 are more stable than others as indicated by the EMSA experiments. RelAp43 could therefore regulate NF-κB dimer formation by potential competition in dimerization between the different pools of NF-κB monomers. RelAp43, like RelA, binds efficiently to p50 and diminishes its processing while it does not affect the processing of the other TAD-containing members of the NF-κB family as shown by cycloheximide and co-IP experiments in cells transfected with anti-RelAp43 siRNA. Although these results do not provide a direct demonstration of the possibility of some exchange of NF-κB dimers, they highly suggest a potential replacement of RelA by RelAp43 in p50-containing dimers, resulting in a higher stability of these complexes.

RelAp43-containing dimers may exhibit a specific transcriptional activity as shown for classical NF-κB dimers [25], [29]. In contrast to p65Δ, p65Δ2, and p65Δ3, RelAp43 does not act as a negative regulator of NF-κB signaling pathway but rather as a co-activator of the pathway. This effect was here particularly exemplified on three different genes involved in innate immune response: *HIAP1*, *IRF1* and *IFN-β* (Fig. 4E). HIAP1, which possesses a RING domain, functions as an E3 ubiquitin ligase [30], [31] and promotes the degradation of caspase-3 and -7 by the proteasome [32], thereby protecting neurons from cell death [33]. RelAp43 could then regulate the balance between cell death and survival. Furthermore, we show that RelAp43 activates the transcription of IRF1, a member of the interferon regulatory transcription factor (IRF) family. Upon induction, IRF1 binds to interferon signalling regulatory elements (*ISRE*) located in promoters of target genes [34] and enhances transcription of IFN-α and -β and of several secondary response genes. IRF1 has also been shown to play roles in regulating apoptosis [35], [36] and is, at least for some viruses, necessary for the antiviral action of IFNs [37]. Interestingly, RelAp43 also exerts a specific induction of the transcription of IFN-β which is dependent on the NF-κB pathway, therefore completing the potential actions of RelAp43 in the innate immune response against viruses.

Viruses possessing negative-single-strand RNA genomes (Order *Mononegavirales*) induce a strong innate immune response and in particular NF-κB-mediated type I interferon response [9], [38]. Among them, lyssavirus are recognized by RIG-I cellular sensor [39] which signals through its interaction with the mitochondrial IPS-1 protein (also referred to as MAVS, Cardif or VISA) [40]. Finally, AP1, IRF3, and NF-κB transcription factors are activated, leading to IFN-β production [11], [41], [42] and inhibition of lyssavirus infection both *in vitro* and *in vivo* [43], [44]. Lyssaviruses have developed several mechanisms to counteract innate immune response [44], [45]: the nucleoprotein has been shown to prevent RIG-I recognition [46], the phosphoprotein P prevents IRF3 phosphorylation, dimerization and nuclear import, it also blocks STATs thus inhibiting interferon expression [47], [48] and the cytoplasmic portion of the G protein trigger survival pathways promoting neuronal survival [49].

The fact that the M protein inhibits NF-κB response probably through direct interaction with RelAp43 further demonstrates the complex role played by this structural protein in viral virulence. M proteins of field lyssavirus isolates have conserved the ability to interact with RelAp43, in contrast to laboratory adapted (PV) or vaccine strains (SAD B-19) which may have lost this feature during virus adaptation to cell culture or during selection while preparing a vaccine. This suggests that the inhibition of the NF-κB response and of the transcription of several RelAp43 dependant genes (*HIAP1*, *IRF1* and *IFN-β*) is critical for the success of the infection and the hence pathogenicity of lyssaviruses. This mechanism of action is independent of those induced by the P which are conserved by the SAD B-19 vaccine strain and which do not modify the NF-κB response [50], [51]. The M protein, by targeting RelAp43 and inhibiting the transcription of HIAP1, a factor protecting neurons from cell death [33], could also regulate the balance between cell death and survival. This indirect action of the M protein on HIAP1 together with those related to the induction of apoptosis [52], [53], [54] further demonstrate the crucial role of the M protein in cell death, although the precise mechanisms which may link RelAp43, HIAP1 and M-mediated apoptosis remain unknown. Therefore, it is tempting to speculate that RelAp43 targeting could be involved in a global reprogramming of the NF-κB pathway to the benefit of lyssavirus infection. A recent study demonstrated the blockade of the NF-κB pathway in rabies-infected neurons, and suggested that one or more viral proteins may directly interact with this pathway [55]. According to our results, we postulate that the M protein could play a role in this blockade, and that its action is mediated by the targeting of RelAp43. Viral manipulations of the NF-κB signaling are a common feature during infections, thus rabies virus would not make exception. Positive strand RNA viruses like the picornaviruses are known to degrade RelA and therefore suppress the innate immune response [56], [57]. DNA viruses of the *Herpesviridae* family also prevent the transcriptional activity of NF-κB [58]. In measles virus, a paramyxovirus, the V protein produced from the P gene has been shown to specifically bind to the RHD of RelA and therefore to suppress NF-κB activity [59]. More generally, other regulatory effects of RelAp43 on cellular processes such as inflammation and oncogenesis may also be expected, as observed in humans with other alternative splicing events of *cRel* and which serves as a marker of tumorigenesis [3].

## Materials and Methods

### Cells and viruses

Human carcinoma epithelial cells (HeLa), human epithelial kidney cells (HEK-293T), and human neuroblastoma cells (IMR5 and SK-S-SH) were cultured as previously described [54]. If indicated, cells were treated with recombinant human TNF-α (R&D systems) at a final concentration of 10 ng/ml and incubated for indicated time at 37°C. When indicated, cycloheximide (Sigma) was added to culture medium at 100 µg/mL for indicated time. Virus infection was performed in 6-well plates during indicated times at 37°C and using different viruses at a multiplicity of infection (MOI) of 1. Thailand virus, referred as Tha (isolate 8743THA), is a field strain of the species rabies virus (RABV) isolated in Thailand from a human bitten by a dog. SAD-B19 virus (SAD) and Pasteur Virus (PV) are vaccine strains of RABV. Field isolates 8720MOK (Mok), 8619NGA (Lag) and 8918FRA (EBLV-1) were used as representative strains of species Mokola virus, Lagos Bat virus and European Bat Lyssavirus-1 (EBLV-1), respectively [60].

### Antibodies

The following antibodies were used: mouse a-V5 antibody (Invitrogen); mouse a-FLAG M2 antibody and rabbit a-FLAG antibody (Sigma); rabbit a-p105/p50, a-p100/p52, a-c-Rel, a-RelB, a-RelA and a-IκBα provided by R Weil; a-RelAp43 polyclonal antibody generated on rabbit immunized with a peptide coding for the specific part of RelAp43 (NH_2_-CGKDFLLSHWNDRFSSVQLRSSGDEDSWAPLQTY-COOH) (Eurogentec); conjugated a-rabbit Alexa 488 (Molecular probes); a-mouse Alexa 555 (Molecular probes); HRP-linked a-mouse antibody and HRP-linked a-rabbit antibody (GE Healthcare).

### RNA isolation, reverse transcription and quantitative real-time PCR

Total RNA was isolated using Nucleospin RNA II kit (Macherey Nagel). Reverse transcription was performed on 2 µg of RNA using Superscript II (Invitrogen) with 2 pmol of oligodT primers (Fermentas) in a final volume of 20 µl. Transcription analysis was performed on 100 ng of total RNA using Taqman Power SYBR Green (Applied Biosystems) in a 7500 instrument (Applied Biosystems) and specific primers (Table S1), following manufacturer instruction. Relative quantification was performed using GAPDH gene as endogenous control gene. Absolute quantification of RelAp43 and RelA was performed using serial dilutions of known quantity of cloned RelA and RelAp43. Results were analyzed using 7500 SDS software version 2 (Applied Biosystems).

### Plasmids

Open reading frame (ORF) coding for M proteins were amplified by reverse transcription-PCR using total RNAs from infected samples [54]. PCRs were carried out with 2 µl of cDNA using primers containing a Gateway cloning site and a matrix specific sequence (Table S1). ORF coding for RelAp43 and RelA were amplified from the cDNA bank that was used for two-hybrid screen using primers indicated in the Supplementary Table S1. Each PCR product was cloned in the pDONR221 vector using Gateway recombinase (Invitrogen). Several final destination vectors were used: yeast two-hybrid vectors pDEST32 and pPC86 in which the Gateway cassette was inserted (Invitrogen), pCDNA3.1N-V5-dest (Invitrogen), pCI-neo-3xFLAG that is a pCI-neo vector (Promega) modified with a 3xFLAG tag (Sigma) in N-terminus of the Gateway cassette (Invitrogen) [61], pM vector (Clontech) in which the Gateway cassette was inserted in order to make the ORF be expressed as a fusion protein with the Gal4 DNA binding domain (Gal4-DB) in N-terminus.

### Western blot

Western blot analysis was performed using NuPAGE gels (Invitrogen). Protein transfer on nitrocellulose membrane was performed using iBlot transfer system (Invitrogen), as indicating by provider. Membranes were saturated for 1 h in PBS-Tween 0.1% with 5% non-fat dried milk. Immunoblotting procedure consisted in incubation for 1 h with indicated primary antibody diluted in 5% dried milk PBS-Tween, then washed three times for 20 min in PBS-Tween, then incubated 1 h with indicated HRP conjugated secondary antibody. Blots were revealed by chemiluminescence (Pierce) and exposure to X-ray films (Amersham) for different time to avoid saturation. Films were digitized and blot relative quantification was performed using Scion Image software.

### Immunofluorescence

Immunofluorescence experiments were performed using as primary antibodies a rabbit anti-FLAG antibody (Sigma) and a mouse anti-V5 (Invitrogen) antibody and as secondary antibodies a conjugated anti-rabbit Alexa 488 and anti- mouse Alexa 555 (Molecular probes) and analysed using Zeiss ApoTome system as described previously [53]. HeLa cells were cultured and transfected with Lipofectamine 2000 in glass Labteck multi-chambers slides. Twenty-four hours post transfection, cells were fixed with cold methanol and permeabilized with acetone.

### EMSA and supershift experiments

Nuclear extracts of HeLa cells were prepared and EMSA were performed as previously described using the κB site derived from the enhancer of the immunoglobulin light chain gene as a probe (Table S1) [62]. For supershift analysis, 100 ng of indicated antibody was incubated to the EMSA reaction mixture. Gel electrophoresis and data collection were performed as described for the EMSA.

### Luciferase reporter gene assays

HEK-293T cells were plated in 96-well plates with 15,000 cells per well under 100 µl of culture medium. After 24 h, cells were transfected with Lipofectamine 2000 (Invitrogen), as recommended by the provider. To measure the NF-κB response, we transfected cells with a mix containing (per well) 160 ng V5-tagged M protein or CAT encoding plasmid, 10 ng of pNF-κB-Luc (Agilent Technologies) coding for firefly luciferase under control of κB sites, and 2 ng of EF1-β-gal (gift from S. Memet, Institut Pasteur) encoding β-galactosidase under control of EF1 promoter insensitive to NF-κB activation. Finally, cells were stimulated or not with recombinant TNF-α at 10 ng/mL (R&D systems). To measure RelA transactivation, we transfected cells with a mix of plasmids composed of 10 ng of pM vector encoding for Gal4-DB fused to RelA, 20 ng of luciferase under control of a Gal4-UAS promoter sequence, and 2 ng of EF1-β-gal. If indicated, we further added 160 ng of plasmid encoding V5-tagged RelAp43, with or without 80 ng of plasmid encoding either V5-tagged M protein or CAT. After 24 h, luciferase and galactosidase activities were measured using Steady-glo and Beta-glo assays (Promega) respectively, as indicated by the provider. Results are the mean of three measures of each activity per transfection and are expressed as the luciferase/beta-galactosidase activity ratio. To measure the late NF-κB response, HEK-293T cells were plated and transfected simultaneously using JetPrime reagent (Polyplus transfection). Transfection mix was composed of 30 ng of pNF-κB-Luc (Agilent Technologies), 3 ng of TK-Renilla luciferase encoding plasmid, 30 ng of plasmid encoding FLAG-tagged M protein or CAT and the indicated amount of 5′-triphosphate RNA. After 24 h, luciferase activities in the lysate were determined using Bright-Glo Luciferase assay system (Promega). The same protocol was used to measure the IFN-β induction, using pIFN-β-Luc, a plasmid encoding firefly luciferase under control of IFN-β promoter.

### Yeast two-hybrid screening

Yeast two-hybrid screen was performed using a human spleen cDNA library cloned in the Gal4-AD pPC86 vector (Invitrogen) and previously established in Y187 yeast strain (Clontech) [63]. We subsequently determined the interaction of various M proteins from different lyssaviruses with RelAp43. ORFs coding for lyssavirus M proteins and RelAp43 were recombined from pDONR221 into pDEST32 and pPC86 yeast two-hybrid vectors, respectively. Yeast cells (AH109 strain, Clontech) were co-transformed with these constructs before platting on a selective medium lacking tryptophan, leucine, histidine and supplemented with 1 mM of 3-aminotriazole (3-AT) (Sigma-Aldrich) to test the interaction-dependent transactivation of the HIS3 reporter gene.

### Immunoprecipitation

Twenty-four hours post transfection, cells were lysed in Ripa buffer (Santa Cruz). For IP of FLAG-tagged proteins, 100 µg of total proteins were incubated with anti-FLAG M2 gel (Sigma) in TNE Triton 1% (w/v) buffer (Tris 25 mM, NaCl 150 mM, antiprotease cocktail (complete Roche) overnight at 4°C on a wheel. Gel was washed with TNE Triton 1% (w/v) buffer. Proteins were eluted directly using loading sample buffer (Invitrogen) and heated 95°C for 10 min. After centrifugation, eluted proteins were analyzed by western blot with the indicated antibody. For IP of non FLAG-tagged proteins, extracts containing 200 µg of total proteins were immunoprecipitated with indicated antibody using washed protein A Sepharose (Sigma). The rest of the protocol is similar as above.

### RNA silencing of RelAp43

HeLa cells were plated out in 6-well plates to reach around 75% confluence the following day. Two small interfering RNAs were designed, one specifically targeting RelAp43 and the other used as a control (Table S1). 20 pmol of siRNA targeting RelAp43 per well were transfected using RNAiMAX Lipofectamine reagent (Invitrogen) according to the manufacturer's instructions. RelAp43 silencing was confirmed by quantitative real-time PCR (Fig. S1). To turn down endogenous RelAp43 expression on infected HeLa cells, control- or antiRelAp43-siRNA were transfected as described above, 3 h post infection.

### Statistical analysis

Single comparisons of data were performed by Student's *t* tests using the GraphPad Prism software.

## Supporting Information

Figure S1 **Decrease of the number of RelAp43 but not RelA mRNA copies in the presence of specific siRNA directed against anti RelAp43 expression.** RelAp43 and RelA mRNA transcription were measured by quantitative RT-PCR in HeLa cells transfected by either a control siRNA (light gray bars) or an anti-RelAp43 siRNA (dark grey bars). For each mRNA, the level of transcription measured in the presence of control siRNA was arbitrary set to 1. The number of RelA mRNA is not modified by anti-RelAp43 siRNA. Results presented here are the mean mRNA level obtained after 3 independent experiments. Significant effects (p<0,05) are indicated by asterisk and error bars indicate standard deviations.
(DOC)

Figure S2 **Transfection of RelAp43 did not induce the production of IκBα.** HeLa cells were transfected with either FLAG-tagged CAT, RelA or RelAp43. After 24 hours, the NF-κB pathway was exogenously activated using 10 ng/mL TNF-α during indicated times. The levels of expression of IκBα, phosphorylated IκBα (IκB-P) and actin were determined by western blot using specific antibodies.
(DOC)

Figure S3 **Luciferase assay of RelA transactivation properties in presence of CAT or RelAp43.** Using luciferase under control of the Gal4 promoter, and RelA fused to the Gal4 DNA Binding domain (named as DB-RelA on the figure). Increasing quantities of RelAp43- or CAT-encoding plasmids were added to the transfection mix in the same conditions as in Figure 3C. (A) Results presented here are the mean luminescence signal obtained after 3 independent experiments. Significant effects (p<0,05) are indicated by asterisk and error bars indicate standard deviations. (B) Transformation of the luminescence units in arbitrary units corresponding to signal with RelAp43/signal with CAT.
(DOC)

Figure S4 **Quantification of RelAp43 mRNA on cells infected with Tha or SAD-B19 virus.** Total RNA were extracted from cells at indicated time post infection. Results are the mean mRNA level obtained after 3 independent experiments. The level of RelAp43 mRNA from cells infected with SAD 1 h p.i. was arbitrary set to 1. Significant effects compared to SAD 1 h p.i. (p<0,05) are indicated by asterisk and error bars indicate standard deviations.
(DOC)

Table S1 **Forward primers (For) and reverse primers (Rev) used in our study.** For Gateway cloning primers, gateway recombination sites are indicated in lower font and ORF specific part in upper font; initiation codon on the forward primer and stop codon on reverse primer are in bold.
(DOC)
