# Supplementary material for: RelAp43, a Member of the NF-κB Family Involved in Innate Immune Response against Lyssavirus Infection
Source: PLoS Pathog. 2012 Dec 13;8(12):e1003060. doi: 10.1371/journal.ppat.1003060 (PMC3521698; doi:10.1371/journal.ppat.1003060)
Supplement: Figure S1 — Decrease of the number of RelAp43 but not RelA mRNA copies in the presence of specific siRNA directed against anti RelAp43 expression. RelAp43 and RelA mRNA transcription were measured by quantitative RT-PCR in HeLa cells transfected by either a control siRNA (light gray bars) or an anti-RelAp43 siRNA (dark grey bars). For each mRNA, the level of transcription measured in the presence of control siRNA was arbitrary set to 1. The number of RelA mRNA is not modified by anti-RelAp43 siRNA. Results presented here are the mean mRNA level obtained after 3 independent experiments. Significant effects (p<0,05) are indicated by asterisk and error bars indicate standard deviations. (DOC) [file ppat.1003060.s001.doc]

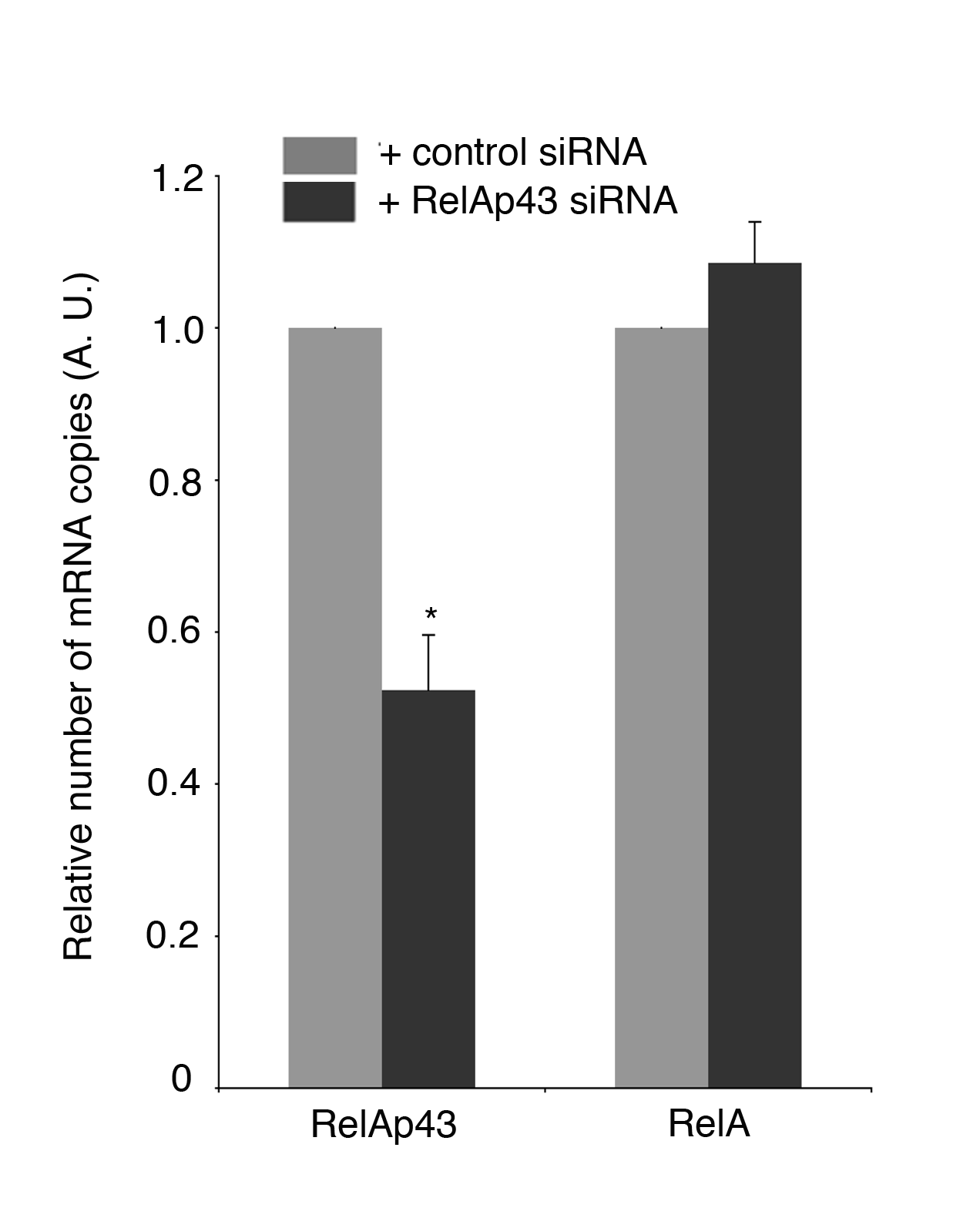


**Figure S1: Decrease of the number of RelAp43 but not RelA mRNA copies in the presence of specific siRNA directed against anti RelAp43 expression.**

RelAp43 and RelA mRNA transcription were measured by quantitative RT-PCR in HeLa cells transfected by either a control siRNA (light gray bars) or an anti-RelAp43 siRNA (dark grey bars). For each mRNA, the level of transcription measured in the presence of control siRNA was arbitrary set to 1. The number of RelA mRNA is not modified by anti-RelAp43 siRNA. Results presented here are the mean mRNA level obtained after 3 independent experiments. Significant effects (p<0,05) are indicated by asterisk and error bars indicate standard deviations.
