# Supplementary material for: RelAp43, a Member of the NF-κB Family Involved in Innate Immune Response against Lyssavirus Infection
Source: PLoS Pathog. 2012 Dec 13;8(12):e1003060. doi: 10.1371/journal.ppat.1003060 (PMC3521698; doi:10.1371/journal.ppat.1003060)
Supplement: Figure S2 — Transfection of RelAp43 did not induce the production of IκBα. HeLa cells were transfected with either FLAG-tagged CAT, RelA or RelAp43. After 24 hours, the NF-κB pathway was exogenously activated using 10 ng/mL TNF-α during indicated times. The levels of expression of IκBα, phosphorylated IκBα (IκB-P) and actin were determined by western blot using specific antibodies. (DOC) [file ppat.1003060.s002.doc]

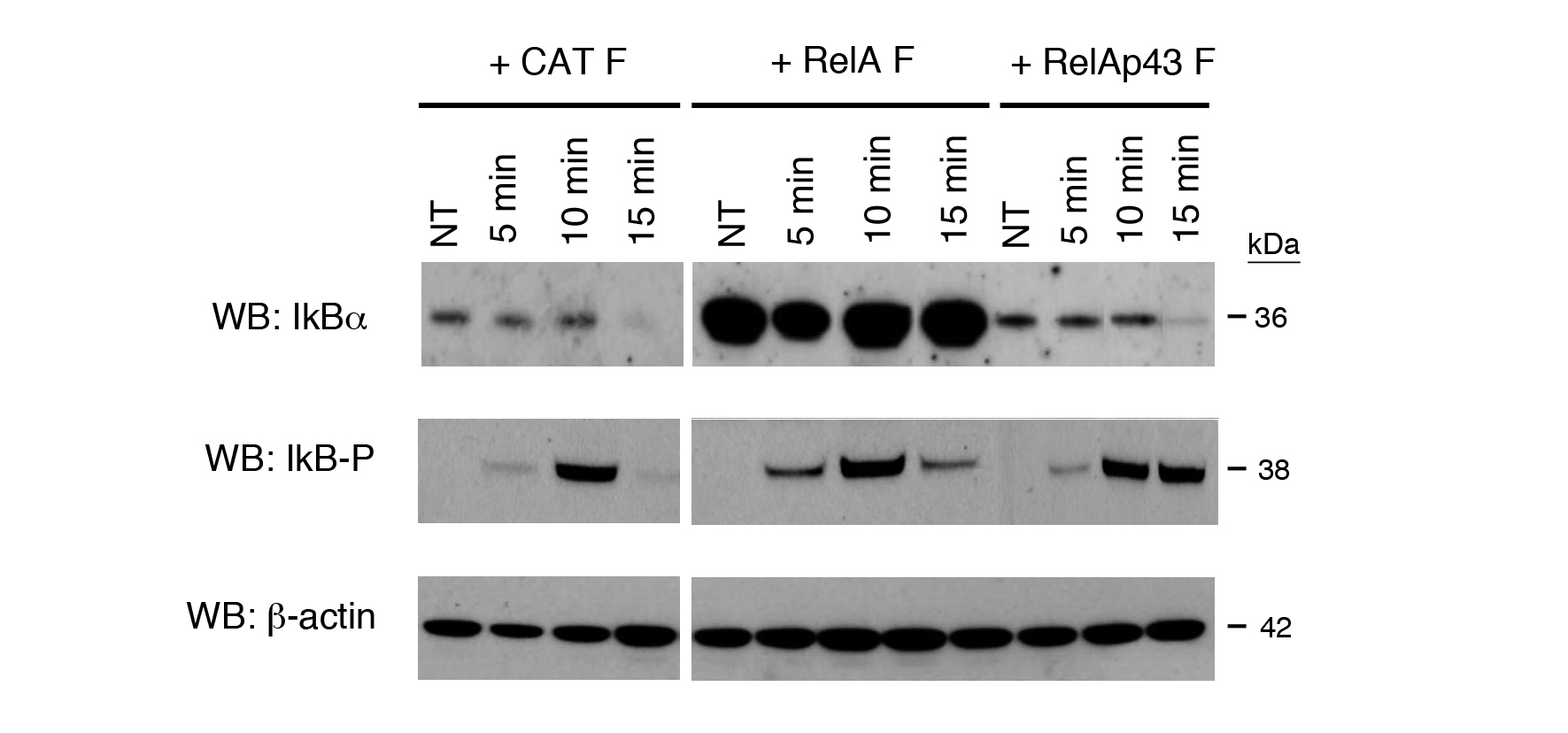


**Figure S2: Transfection of RelAp43 did not induce the production of IκBα.**

HeLa cells were transfected with either FLAG-tagged CAT , RelA or RelAp43. After 24 hours, the NF-κB pathway was exogenously activated using 10 ng/mL TNF-α during indicated times. The levels of expression of IκBα, phosphorylated IκBα (IκB-P) and actin were determined by western blot using specific antibodies.
