# Supplementary material for: RelAp43, a Member of the NF-κB Family Involved in Innate Immune Response against Lyssavirus Infection
Source: PLoS Pathog. 2012 Dec 13;8(12):e1003060. doi: 10.1371/journal.ppat.1003060 (PMC3521698; doi:10.1371/journal.ppat.1003060)
Supplement: Figure S3 — Luciferase assay of RelA transactivation properties in presence of CAT or RelAp43. Using luciferase under control of the Gal4 promoter, and RelA fused to the Gal4 DNA Binding domain (named as DB-RelA on the figure). Increasing quantities of RelAp43- or CAT-encoding plasmids were added to the transfection mix in the same conditions as in Figure 3C. (A) Results presented here are the mean luminescence signal obtained after 3 independent experiments. Significant effects (p<0,05) are indicated by asterisk and error bars indicate standard deviations. (B) Transformation of the luminescence units in arbitrary units corresponding to signal with RelAp43/signal with CAT. (DOC) [file ppat.1003060.s003.doc]

**A**


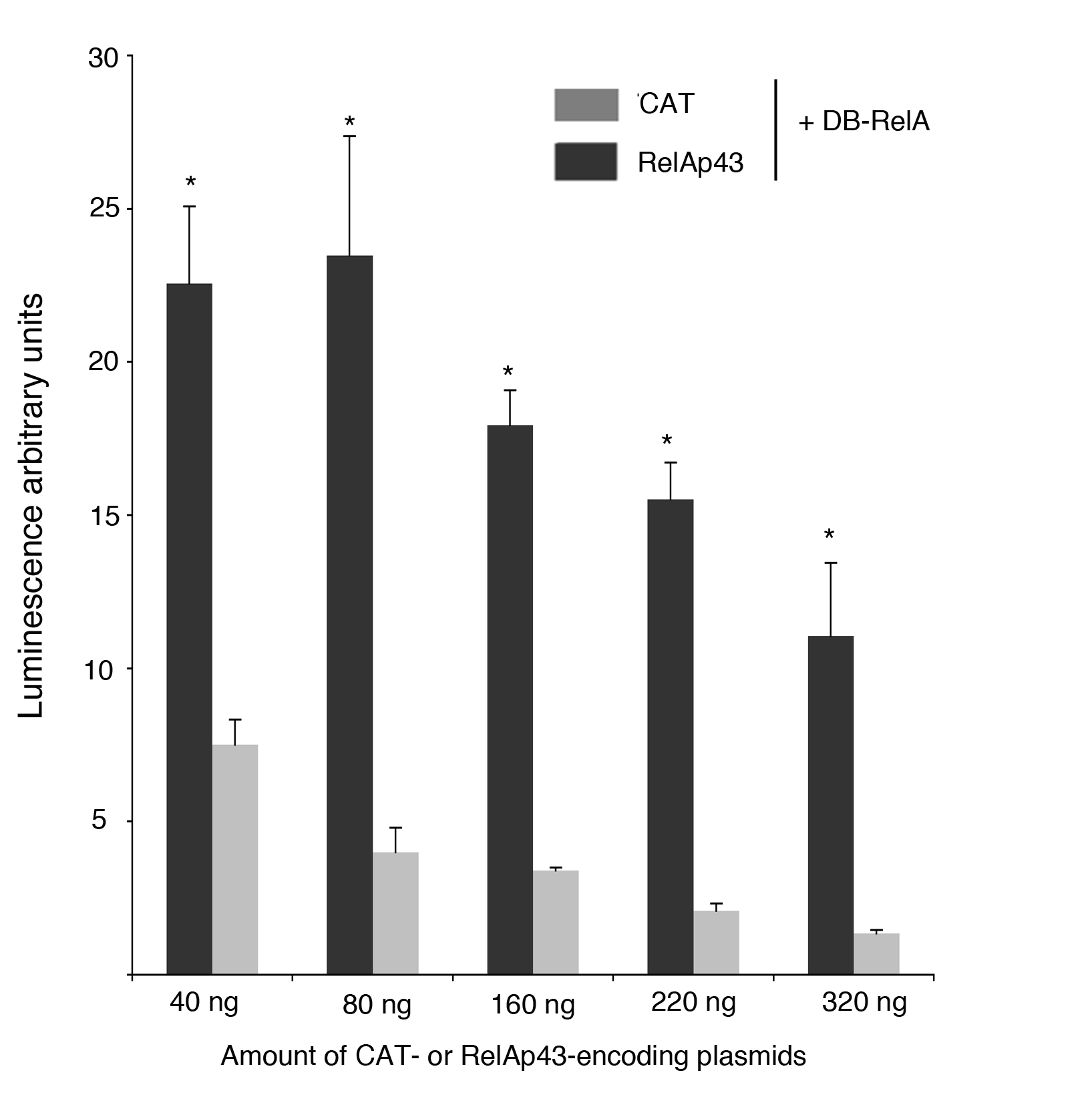


**B**


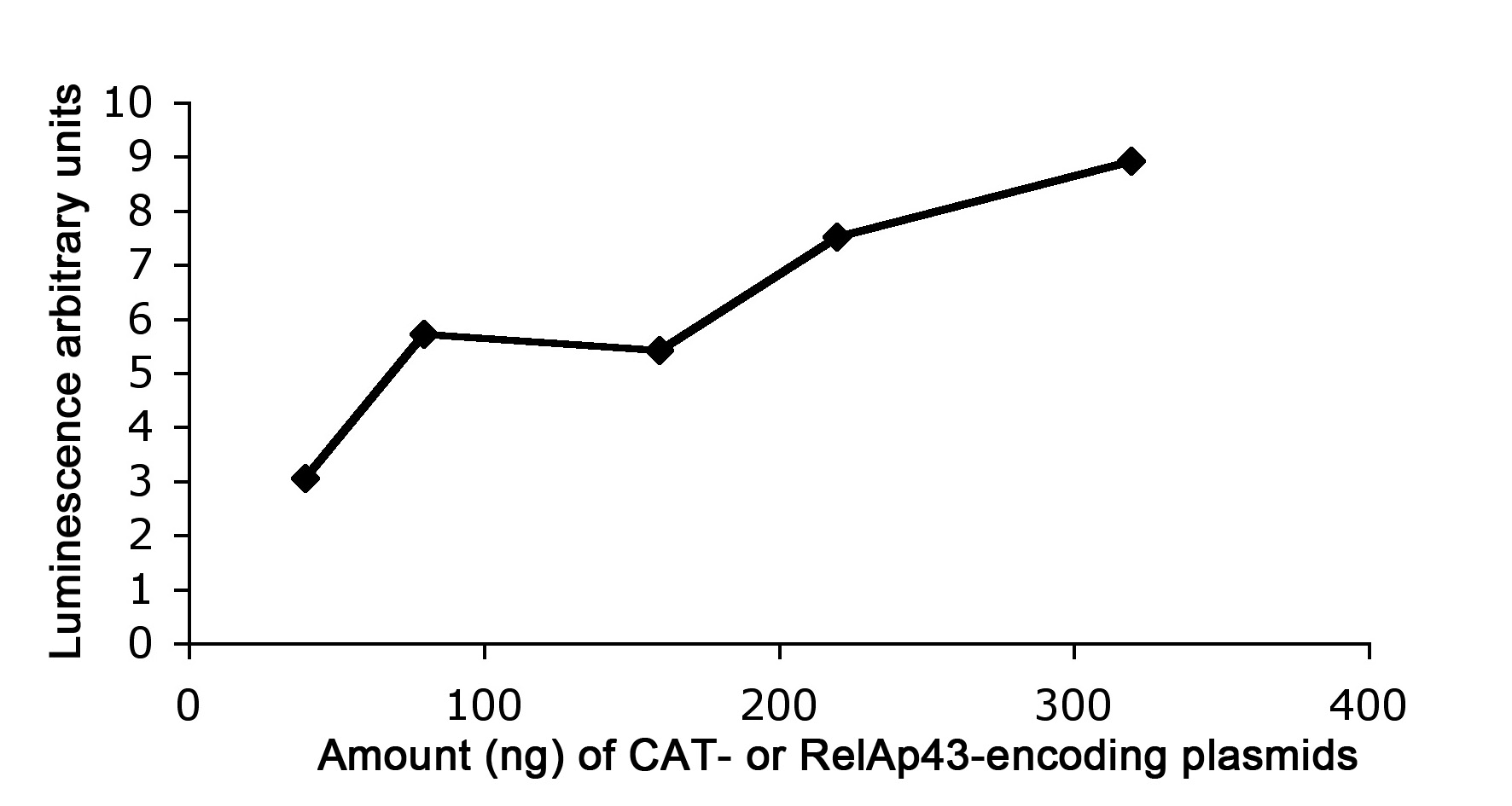


**Figure S3: Luciferase assay of RelA transactivation properties in presence of CAT or RelAp43**. Using luciferase under control of the Gal4 promoter, and RelA fused to the Gal4 DNA Binding domain (named as DB-RelA on the figure). Increasing quantities of RelAp43- or CAT-encoding plasmids were added to the transfection mix in the same conditions as in Figure 3C. (A) Results presented here are the mean luminescence signal obtained after 3 independent experiments. Significant effects (p<0,05) are indicated by asterisk and error bars indicate standard deviations. (B) Transformation of the luminescence units in arbitrary units corresponding to signal with RelAp43/signal with CAT.
