# Supplementary material for: RelAp43, a Member of the NF-κB Family Involved in Innate Immune Response against Lyssavirus Infection
Source: PLoS Pathog. 2012 Dec 13;8(12):e1003060. doi: 10.1371/journal.ppat.1003060 (PMC3521698; doi:10.1371/journal.ppat.1003060)
Supplement: Figure S4 — Quantification of RelAp43 mRNA on cells infected with Tha or SAD-B19 virus. Total RNA were extracted from cells at indicated time post infection. Results are the mean mRNA level obtained after 3 independent experiments. The level of RelAp43 mRNA from cells infected with SAD 1 h p.i. was arbitrary set to 1. Significant effects compared to SAD 1 h p.i. (p<0,05) are indicated by asterisk and error bars indicate standard deviations. (DOC) [file ppat.1003060.s004.doc]

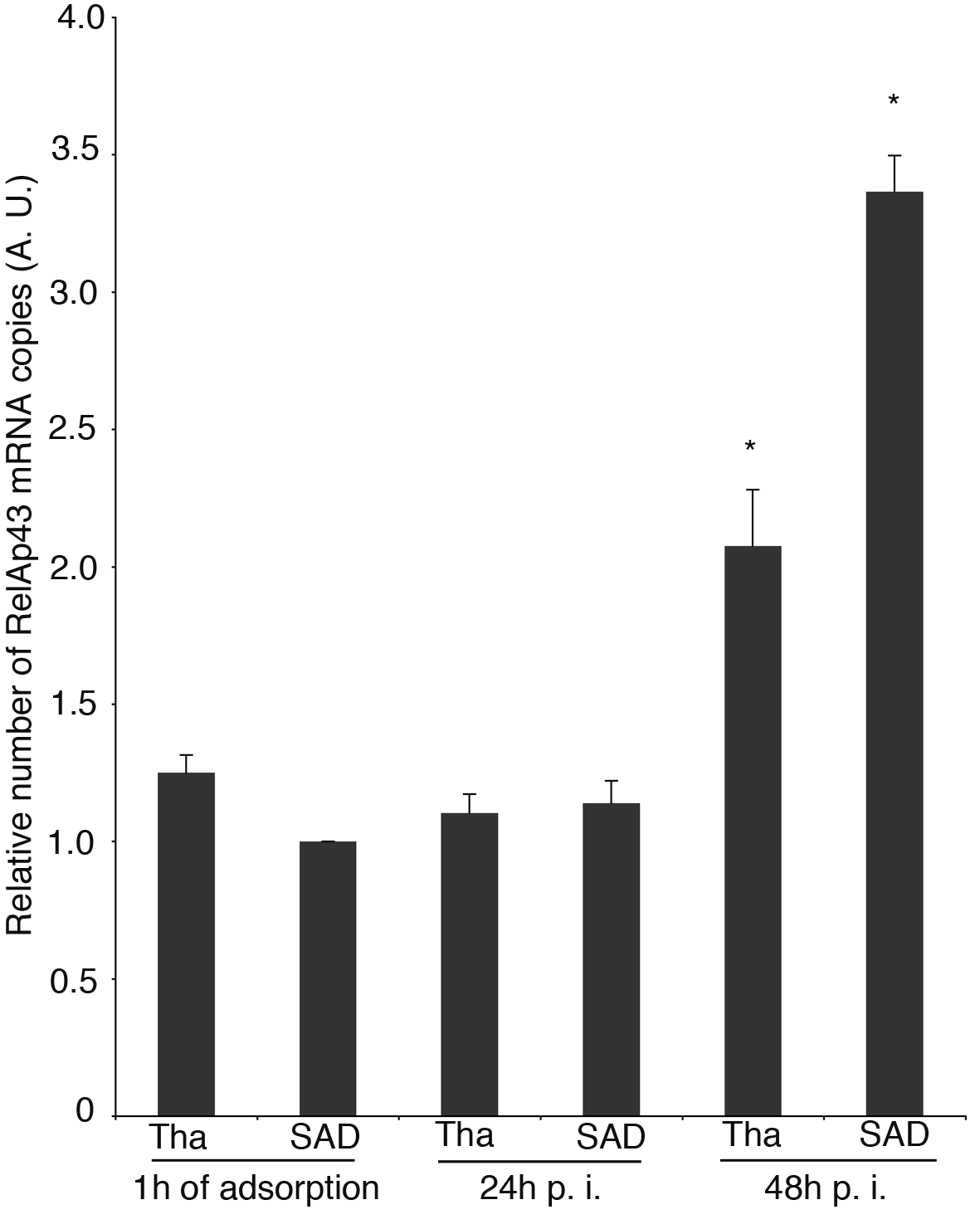


**Figure S4: Quantification of RelAp43 mRNA on cells infected with Tha or SAD-B19 virus.**

Total RNA were extracted from cells at indicated time post infection. Results are the mean mRNA level obtained after 3 independent experiments. The level of RelAp43 mRNA from cells infected with SAD 1 h p.i. was arbitrary set to 1. Significant effects compared to SAD 1 h p.i. (p<0,05) are indicated by asterisk and error bars indicate standard deviations.
