# Supplementary material for: RelAp43, a Member of the NF-κB Family Involved in Innate Immune Response against Lyssavirus Infection
Source: PLoS Pathog. 2012 Dec 13;8(12):e1003060. doi: 10.1371/journal.ppat.1003060 (PMC3521698; doi:10.1371/journal.ppat.1003060)
Supplement: Table S1 — Forward primers (For) and reverse primers (Rev) used in our study. For Gateway cloning primers, gateway recombination sites are indicated in lower font and ORF specific part in upper font; initiation codon on the forward primer and stop codon on reverse primer are in bold. (DOC) [file ppat.1003060.s005.doc]

| **ORF for Gateway cloning** |  | **Primer sequence 5' to 3'** | **Accession Number in Viral-**  **ORFeome** |
| --- | --- | --- | --- |
| M-Mok | For | ggggacaactttgtacaaaaaagttggc**atg**AATTTCCTCAAGAAAATGATCAAGAGCT | 81 |
|  | Rev | ggggacaactttgtacaagaaagttgg**tta**CTCTAATAAAAGTGAGGTGTTTTCATCA |  |
| M-PV | For | ggggacaactttgtacaaaaaagttggc**atg**AACTTTCTACGTAAGATAGTGAAAAATTGCAG | 921 |
|  | Rev | ggggacaactttgtacaagaaagttgg**tta**TTCTAGAAGCAGAGAGGAATCTTTGTCCTCTT |  |
| M-Lag | For | ggggacaactttgtacaaaaaagttggc**atg**AATTTCCTGAGGAAGATAGTGAAGAATT | 913 |
|  | Rev | ggggacaactttgtacaagaaagttgg**tta**TTCCAACAGAAGTGAAGTGTTCTCATCT |  |
| M-Tha | For | ggggacaactttgtacaaaaaagttggc**atg**AACTTTCTACGCAAAATCGTGAAAAACT | 929 |
|  | Rev | ggggacaactttgtacaagaaagttgg**tta**TTCTAGGAGCAGGGAAGAGTCTTTATCT |  |
| M-EBL1a | For | ggggacaactttgtacaaaaaagttggc**atg**AATATCATTCGGAAAATTGTTAAGAGCT | 915 |
|  | Rev | ggggacaactttgtacaagaaagttgg**tta**TTCAAGGAGCAAGGAAGTGTTCTTGTCT |  |
| RelA | For | ggggacaactttgtacaaaaaagttggc**atg**GACGAACTGTTCCCCC | / |
|  | Rev | ggggacaactttgtacaagaaagttgg**tta**GGAGCTGATCTGACTCA |  |
| RelAp43 | For | ggggacaactttgtacaaaaaagttggc**atg**GACGAACTGTTCCCCC | / |
|  | Rev | ggggacaactttgtacaagaaagttgg**tca**GTAGGTCTGTAATGGGGC |  |
| CAT | For | ggggacaactttgtacaaaaaagttggc**atg**GAGAAGAAGATCACTGGAT | / |
|  | Rev | ggggacaactttgtacaagaaagttgg**tta**CGCCCCGCCCTGCCA |  |
| **EMSA experiments** |  | **Primer sequence 5' to 3'** |  |
| EMSA probe | For | gatcatggggaatcccca |  |
|  | Rev | gatctggggattccccat |  |
| **Real-time PCR primers** |  | **Primer sequence 5' to 3'** |  |
| *RelAp43* | For | GAGCAGTGGAGATGAAGACTCTTG |  |
|  | Rev | CCATCCTTTCAAAGCCTCTGAT |  |
| *RelA* | For | GGCCCCCCACACAACTG |  |
|  | Rev | CCCCTGTCACTAGGCGAGTTAT |  |
| *Bcl2* | For | TCAAGTGTTCCGCGTGATTG |  |
|  | Rev | TTATTGGATGTGCTTTGCATTCTT |  |
| *XIAP* | For | TGTTTTGGGCCGGAATCTTA |  |
|  | Rev | TTTGTTGAATTTGGGAAATTCCTAT |  |
| *c-FLIP* | For | GTGGAGACCCACCTGCTCA |  |
|  | Rev | GGACACATCAGATTTATCCAAATCC |  |
| *HIAP1* | For | GGCATTGTACTAATACCGGGAACA |  |
|  | Rev | CTTGCCTCAGCCTGGGACT |  |
| *HIAP2* | For | GCTTGTTCAGTGGTTCTTACTCCA |  |
|  | Rev | GGTTAGTCCTCGATGAAGAGATGTC |  |
| *MCP1* | For | TCGCCTCCAGCATGAAAGTC |  |
|  | Rev | GGCATTGATTGCATCTGGC |  |
| *IRF1* | For | CCCCAGAAAAGCATAACACCA |  |
|  | Rev | TTTCGCTTAGTGCAGAGCCA |  |
| *IFN-b* | For | TGACATCCCTGAGGAGATTAAGC |  |
|  | Rev | GCGTCCTCCTTCTGGAACTG |  |
| *GAPDH* | For | TGGAAGGACTCATGACCACAGT |  |
|  | Rev | CAGTCTTCTGGGTGGCAGTGA |  |
|  |  |  |  |
|  |  |  |  |
| **RNA silencing** | **Primer sequence 5' to 3'** | |  |
| aRelAp43 siRNA | CCCAAGCCAGGUAAGGAUUUCCUUU | |  |
| Control siRNA | CCCACCGAUGGAGGACUUUCAAUUU | |  |

**Table S1: Forward primers (For) and reverse primers (Rev) used in our study.**

For Gateway cloning primers, gateway recombination sites are indicated in lower font and ORF specific part in upper font; initiation codon on the forward primer and stop codon on reverse primer are in bold.
